# Postnatal Maxillofacial “Developing” Decellularized Extracellular Matrix Orchestrates Hierarchical Cross-Organ Regeneration via Macrophage Integrin αvβ5-Mediated Efferocytosis-Driven Developmental Recapitulation

**DOI:** 10.34133/research.1234

**Published:** 2026-04-15

**Authors:** Ruidi Xia, Junlong Xue, Xinyu Liu, Mixiao Gui, Linjun Zhang, Yihua Cai, Zhengjie Shan, Guanqi Liu, Xuran Liao, Zhuofan Chen, Jieyun Xu, Zetao Chen

**Affiliations:** ^1^ Hospital of Stomatology, Guanghua School of Stomatology, Sun Yat-sen University and Guangdong Provincial Key Laboratory of Stomatology, Guangzhou 510055, China.; ^2^ Guangdong Research Center for Dental and Cranial Rehabilitation and Material Engineering, Guangzhou 510055, China.; ^3^ Foshan Stomatology Hospital and School of Medicine, Foshan University, Foshan 528000, China.

## Abstract

Decellularized extracellular matrix (dECM) leverages native architecture and bioactive components for tissue regeneration, yet its therapeutic efficacy is constrained by donor tissue maturity. While mature-tissue-derived dECM (Mat-dECM) lacks developmental signals, developmental-stage dECM (Dev-dECM) retains these cues but is limited by scarce sources and poor adaptability to adult environments. Here, we proposed postnatal maxillofacial odontogenic tissues as a novel Dev-dECM (pDev-dECM) source. Leveraging its unique trans-stage development—spanning embryonic crown formation to postnatal periodontal maturation—pDev-dECM balances developmental potency with adult environment adaptability. Multiomics comparative analysis revealed that pDev-dECM is enriched in arginylglycylaspartic-acid-containing proteins, which activate macrophage integrin αvβ5-mediated efferocytosis. This mechanism drives macrophage polarization toward regenerative M2 phenotypes, ensures adaptability to the adult environment, and reprograms mesenchymal stem cells to orchestrate developmental recapitulation. Consequently, pDev-dECM not only enabled in situ hierarchical regeneration of the periodontal complex but also facilitated cross-organ hierarchical regeneration in skin and muscle defect models. These findings demonstrate that pDev-dECM exerts spatiotemporal control over developmental recapitulation, establishing a universal biomaterial paradigm for multiscale tissue reconfiguration.

## Introduction

Decellularized extracellular matrix (dECM) preserves native bioactive components (e.g., collagens and proteoglycans) and topological architecture, offering precise microenvironmental cues to stem cells and immune cells [1–5]. However, current strategies predominantly rely on homologous mature-tissue-derived dECM (Mat-dECM) of large mammals to target organ repair [2,3,6–10]. While Mat-dECM benefits from high yield and host adaptability, its terminally differentiated nature lacks the dynamic developmental signals required for complex hierarchical regeneration. Organ regeneration involves orchestrated processes—such as mesenchymal condensation and morphogenesis—governed by signaling networks such as Wnt, Hedgehog, and efferocytosis [11–20]. Consequently, Mat-dECM, which supports only basic repair, is incapable of reinitiating these developmental cascades to reconstruct multilayered architectures [11–13].

To overcome these limitations, developmental-stage dECM (Dev-dECM) has emerged as a potent alternative. Comparative analyses indicate that embryonic Dev-dECM retains the molecular foundation (e.g., hyaluronic acid and arginylglycylaspartic acid [RGD]-related proteins) necessary to remodel osteogenic microenvironments and stimulate angiogenesis [3,12,13]. However, the clinical translation of embryonic Dev-dECM is severely hindered by donor scarcity and poor adaptability to the adult host immune environment [21,22].

Here, we propose postnatal maxillofacial odontogenic tissues as a novel “developing” dECM (pDev-dECM) source to bridge this gap. Unlike most organs that complete morphogenesis embryonically, teeth and periodontal complexes exhibit a unique trans-stage development: crown formation is embryonic, while root and periodontal maturation occur postnatally [23,24]. This spatiotemporal paradigm, combined with the donor abundance from the mammalian diphyodont system [25–27], endows pDev-dECM with dual advantages: retention of developmental signaling and adaptability to adult environments.

To validate this concept, we utilized mandibular tissues from 20-d postnatal rats (active developmental phase) to prepare pDev-dECM, comparing it against Mat-dECM. Proteomic and single-cell transcriptomic analyses revealed that pDev-dECM reactivates developmental processes via macrophage-mediated matrix-instructive signaling. We further validated its efficacy not only in the complex hierarchical regeneration of periodontal defects but also in cross-organ skin (ectodermal) and muscle (mesodermal) models. This study identifies a unique “developing” matrix with intrinsic organogenic plasticity that drives architecturally ordered regeneration, establishing pDev-dECM as a universal biomaterial platform for multiscale tissue reconfiguration.

## Results

### Fabrication and characterization of pDev-dECM and Mat-dECM

**Fig. 1.**
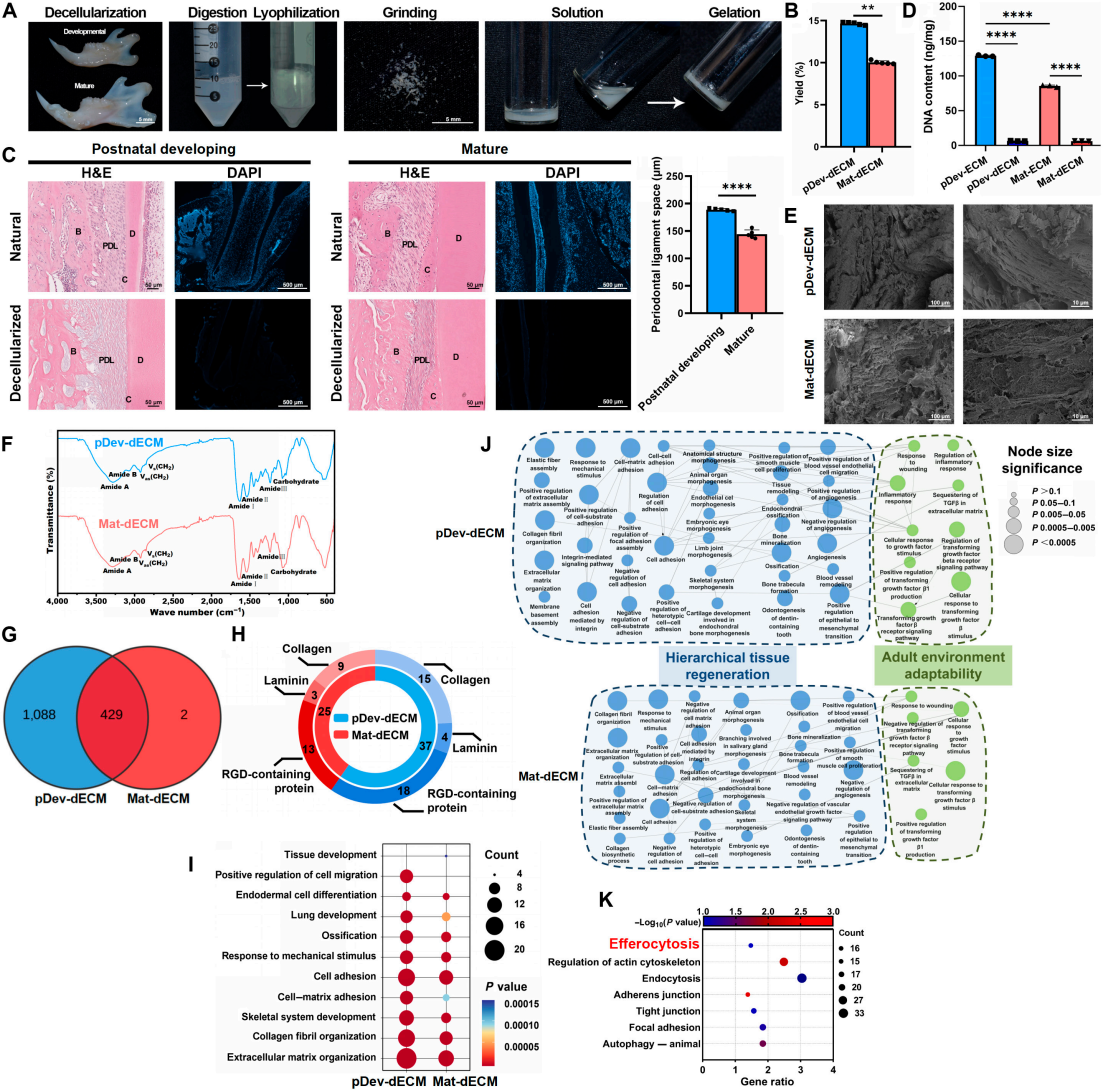
Fabrication and characterization of postnatal developmental-stage decellularized extracellular matrix (pDev-dECM) and mature-tissue-derived decellularized extracellular matrix (Mat-dECM). (A) Schematic workflow for pDev-dECM and Mat-dECM preparation, involving mandible decellularization, enzymatic digestion, lyophilization, pulverization, reconstitution, and gelation. (B) Quantification of dECM yield comparing pDev-dECM and Mat-dECM (*n* = 5 per group). (C) Histological validation of the natural and decellularized mandible using hematoxylin and eosin (H&E) (morphology) and 4′,6-diamidino-2-phenylindole (DAPI) (nuclear clearance) staining was followed by quantitative analysis of periodontal ligament space width in postnatal developing and mature groups (*n* = 5 per group). (D) Quantitative analysis of residual DNA content in native and decellularized mandibles (*n* = 3 per group). (E) Scanning electron microscopy (SEM) images characterizing the microstructure of pDev-dECM and Mat-dECM hydrogels. (F) Fourier transform infrared (FTIR) spectra of pDev-dECM and Mat-dECM. (G) Venn diagram comparing the proteomic composition of pDev-dECM and Mat-dECM (*n* = 3 per group). (H) Classification of identified ECM proteins acting as ligands for specific receptors in both groups (*n* = 3 per group). (I and J) Gene Ontology biological process (GO-BP) enrichment analysis of ECM proteins bubble diagram and network diagram of the classification of various enriched biological events of pDev-dECM and Mat-dECM. (K) Kyoto Encyclopedia of Genes and Genomes (KEGG) enrichment analysis highlighting pDev-dECM-specific proteins enriched in efferocytosis-related signaling pathways. PDL, periodontal ligament; B, alveolar bone; C, cementum; D, dentin. Data were presented as means ± SD. Significance was determined by unpaired 2-tailed Student’s *t* test or Mann–Whitney *U* test (2 groups) and one-way ANOVA with Tukey’s post hoc test (multiple groups). *P < 0.05, **P < 0.01, ***P < 0.001, and ****P < 0.0001.

To achieve a strategic equilibrium between bioinductive potential and biocompatibility [28,29], we optimized a 1% Triton X-100/1% sodium dodecyl sulfate decellularization protocol. Posttreatment, mandibles retained anatomical morphology while appearing translucent white. Hydrogels were subsequently prepared via mechanical grinding, acid extraction, and pepsin digestion. Upon pH neutralization and 37 °C incubation, the pregel solutions formed stable hydrogels (Fig. 1A). Yield quantification indicated higher matrix efficiency in pDev-dECM (Fig. 1B), suggesting intrinsic developmental ECM consolidation that may mitigate embryonic donor limitations.

Histological analysis of native developing periodontium revealed immature features, widened ligament spaces, and loose collagen, distinct from the aligned, mature fiber bundles of the mature group (Fig. 1C). Postdecellularization hematoxylin and eosin (H&E) and 4′,6-diamidino-2-phenylindole (DAPI) fluorescence confirmed complete nuclear clearance and collagen preservation. Residual DNA fell below the accepted threshold of 50 ng/mg (Fig. 1D) [30], confirming successful decellularization. Notably, native pDev-ECM contained higher DNA content, reflecting proliferative developmental populations.

Scanning electron microscopy (SEM) revealed a rough, lamellar topology in both hydrogels (Fig. 1E). Fourier transform infrared (FTIR) spectroscopy (Fig. 1F) confirmed the retention of characteristic collagen peaks, including amide A (N–H stretching), amide B (amide A/I coupling), amide I (C═O stretching/N–H bending), amide II (N–H bending/C–N stretching), and amide III (N–H bending/C–N interactions), as well as proteoglycan-specific peaks at 1,140 cm^−1^.

### pDev-dECM exhibits excellent dual effect in developmental recapitulation to achieve hierarchical tissue regeneration and adaptability to the adult environment

Comprehensive proteomic profiling revealed distinct compositional differences between pDev-dECM and Mat-dECM, despite both preserving bioactive components [1,3,4]. Principal components analysis (PCA) confirmed dataset reliability (Fig. S1A), while heatmaps showed elevated protein expression in pDev-dECM (Fig. S1B). Quantitatively, pDev-dECM contained 1,517 proteins (1,088 unique) versus 431 in Mat-dECM (2 unique) (Fig. 1G), dominating across all ECM subclasses (Fig. S1C). Since ECM proteins regulate cell behavior via specific receptors [31–33], we categorized the identified proteins based on their cognate receptors. As shown in Fig. 1H, pDev-dECM possessed a substantially higher diversity of receptor-binding ligands, particularly RGD-containing proteins, than Mat-dECM. Core matrisome levels are detailed in Fig. S1D.

Gene Ontology biological process (GO-BP) enrichment analysis indicated broader and more significant enrichment of tissue regeneration processes in pDev-dECM (Fig. 1I). To synthesize the functional landscape, we categorized these biological processes into hierarchical tissue regeneration (encompassing mesenchymal condensation and multiple tissue regeneration) and adult environment adaptability (encompassing immune regulation and response to stimulus) (Fig. 1J and Fig. S1E). Notably, pDev-dECM exhibited substantially more events driving hierarchical tissue regeneration, including mesenchymal condensation—a prerequisite for organogenesis [14,16–20]. Crucially, pDev-dECM maintained adult environment adaptability comparable to Mat-dECM but with a superior total number of enriched events, suggesting greater functional potency.

Kyoto Encyclopedia of Genes and Genomes (KEGG) enrichment analysis of the pDev-dECM-unique proteome highlighted enriched pathways including efferocytosis, cytoskeleton regulation, endocytosis, and adhesion (Fig. 1K). Protein mapping (Fig. S1F) revealed that pDev-dECM is enriched in components regulating all efferocytosis stages: recognition/sensing (smell), phagocytosis/cytoskeletal remodeling (eat), and digestion/metabolic adaptation (digest) (Fig. S1G), suggesting a multitarget mechanism to enhance apoptotic cell clearance.

Given macrophages’ pivotal role as the primary responders to implanted biomaterials [34–36] and their function as ECM sentinels that translate microenvironmental cues into regenerative commands [37], we specifically subclustered this population to investigate dECM-mediated macrophage regulation. This analysis aimed to clarify how pDev-dECM mimics the developmental immune microenvironment to orchestrate developmental recapitulation.

### The underlying mechanism of pDev-dECM’s developmental recapitulation lies in the regulation of macrophage-mediated efferocytosis

**Fig. 2.**
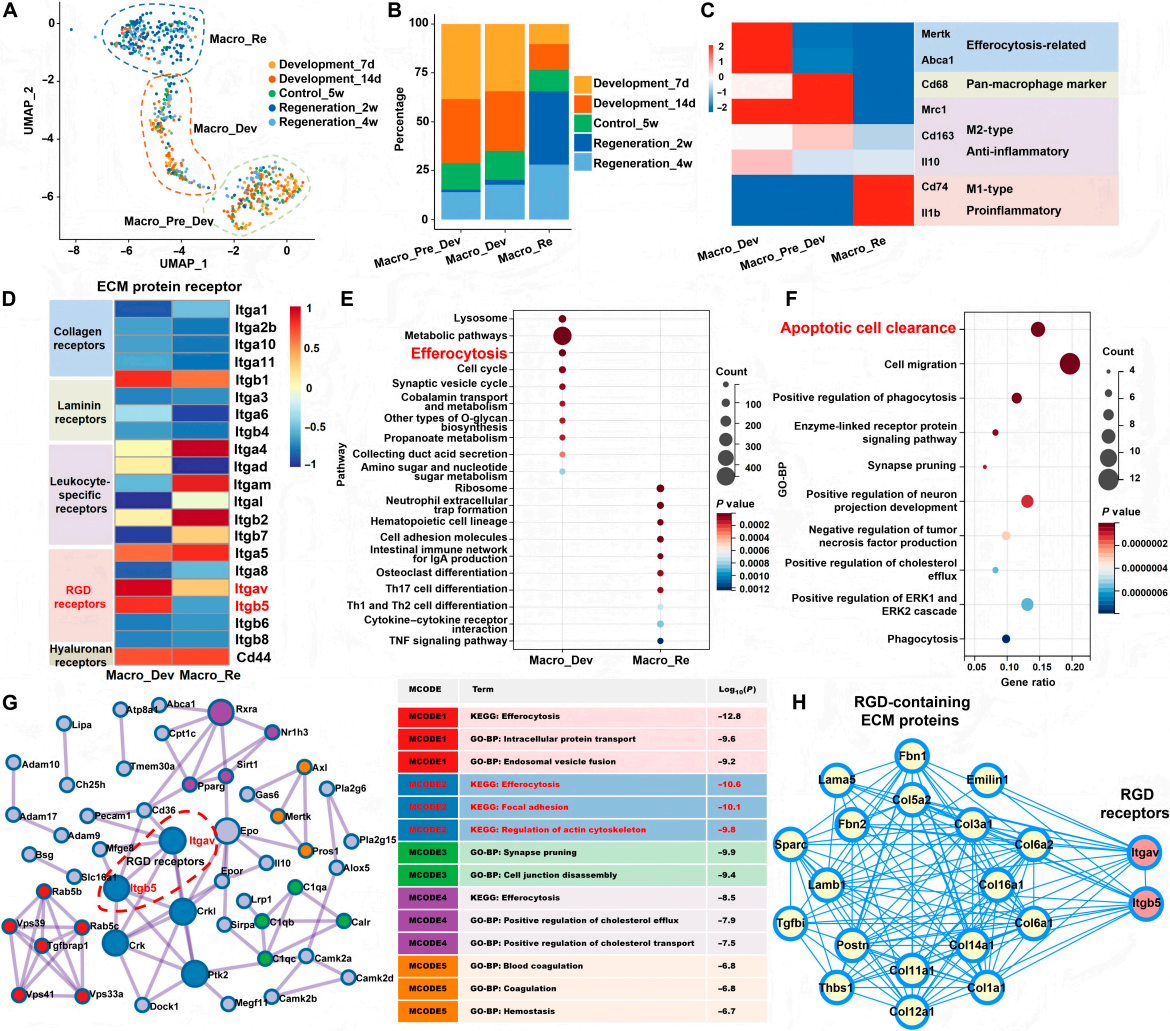
Single-cell profiling of periodontal tissue development, maturation, and repair reveals extracellular matrix (ECM) regulatory roles. (A) Uniform Manifold Approximation and Projection (UMAP) atlas of macrophage subsets across all stages. (B) Macrophage subset distribution per stage. (C) Expression heatmap of pan-macrophage, M1/M2, and efferocytosis markers. (D) ECM receptor expression in Macro_Re versus Macro_Dev subsets. (E) KEGG enrichment analysis of differentially expressed genes (DEGs) between Macro_Re and Macro_Dev. (F) GO-BP analysis of efferocytosis-enriched genes in Macro_Dev. (G) Molecular complex detection (MCODE) clustering of up-regulated efferocytosis genes in Macro_Dev. The network identifies integrin signaling–cytoskeletal remodeling modules, highlighting the co-clustering of receptors (*Itgav* and *Itgb5*) with effectors (*Ptk2*, *Crk*, and *Dock1*) essential for actin reorganization. Table lists cluster-specific terms (colors match clusters). (H) Protein–protein interaction (PPI) network linking arginylglycylaspartic acid (RGD) receptors (*Itgav* and *Itgb5*) to pDev-dECM RGD ligands.

To dissect dynamic ECM-immune cross-talk [31–33,38], we profiled macrophage dynamics via single-cell RNA sequencing (scRNA-seq) across periodontal development (postnatal days 7 and 14), maturity (5 weeks), and repair (2 and 4 weeks postdefect). The resulting atlases (Fig. 2A to C) revealed distinct stage-specific subsets. During development, macrophages segregated into a precursor-like M2 subset (Macro_Pre_Dev) and a functional effector subset (Macro_Dev). While both shared high expression of *Mrc1*, Macro_Dev was specifically distinguished by the up-regulation of essential efferocytosis machinery (*Mertk* and *Abca1*), marking the acquisition of functional clearance capacity. Conversely, the repair period was dominated by a regenerative subset (Macro_Re) characterized by M1 and inflammatory markers (*Cd74* and *Il1b*). Mature populations remained low and balanced, reflecting a stabilized microenvironment.

We next investigated whether these phenotypic shifts were driven by differential ECM receptor expression. While 2 major receptor classes (integrin-family genes and *Cd44*) were detected, specific profiling (Fig. 2D) revealed a striking divergence: Macro_Dev up-regulated RGD-binding receptors (*Itgav* and *Itgb5*), whereas Macro_Re favored leukocyte-specific integrins (*Itga4*, *Itgam*, *Itgb2*, and *Itgb7*) [32]. This specific integrin αvβ5 activation mirrors our proteomic findings (Fig. 1H) of abundant RGD-containing ligand proteins in pDev-dECM, suggesting that the microenvironment is intrinsically primed to activate Macro_Dev via the RGD–integrin axis.

To elucidate downstream consequences [39], KEGG and GO-BP analyses (Fig. 2E and F) confirmed the synchronous enrichment of lysosome, metabolic pathways, and efferocytosis in Macro_Dev. This profile indicates active engulfment coupled with intracellular degradation and nutrient processing, contrasting with the inflammatory signaling observed in Macro_Re. Crucially, molecular complex detection (MCODE) analyses (Fig. 2G) identified a functional cluster involving *Itgav*/*Itgb5* and downstream effectors (*Ptk2* and *Crk*/*Crkl*-*Dock1*). Dot plot validation (Fig. S2) confirmed the specific, concomitant expression of these components within the Macro_Dev subset, indicating direct activation of the adhesion–cytoskeletal remodeling cascade driving engulfment. Furthermore, protein–protein interaction analysis (PPI) (Fig. 2H) corroborated interactions between RGD-containing ECM proteins in the pDev-dECM proteome and their cognate receptors, *Itgav* and *Itgb5*.

Collectively, pDev-dECM utilizes enriched RGD motifs to engage the integrin αvβ5 axis, activating the cytoskeletal machinery for efferocytosis and polarizing macrophages toward a regenerative phenotype distinct from inflammatory response triggered by mature or repair-phase ECM.

### RGD-enriched pDev-dECM targets integrin αvβ5 to reprogram macrophage-mediated efferocytosis in vitro

To validate whether developmental signals, specifically the RGD-enriched proteins (e.g., *Fn1* and *Postn*) and efferocytosis–regulatory factors (e.g., *Lrp1*, *Alox15*, and *Crkl*), can be transmitted to adult macrophages in vitro, we cocultured RAW 264.7 cells with dECM hydrogels. Cell Counting Kit-8 assays confirmed biocompatibility at 48 h (Fig. S3A). Transcriptomic sequencing at 24 h revealed distinct regulatory patterns. PCA and heatmap clustering (Fig. S3B and C) confirmed that pDev-dECM induces specific transcriptomic reprogramming distinct from Mat-dECM.

**Fig. 3.**
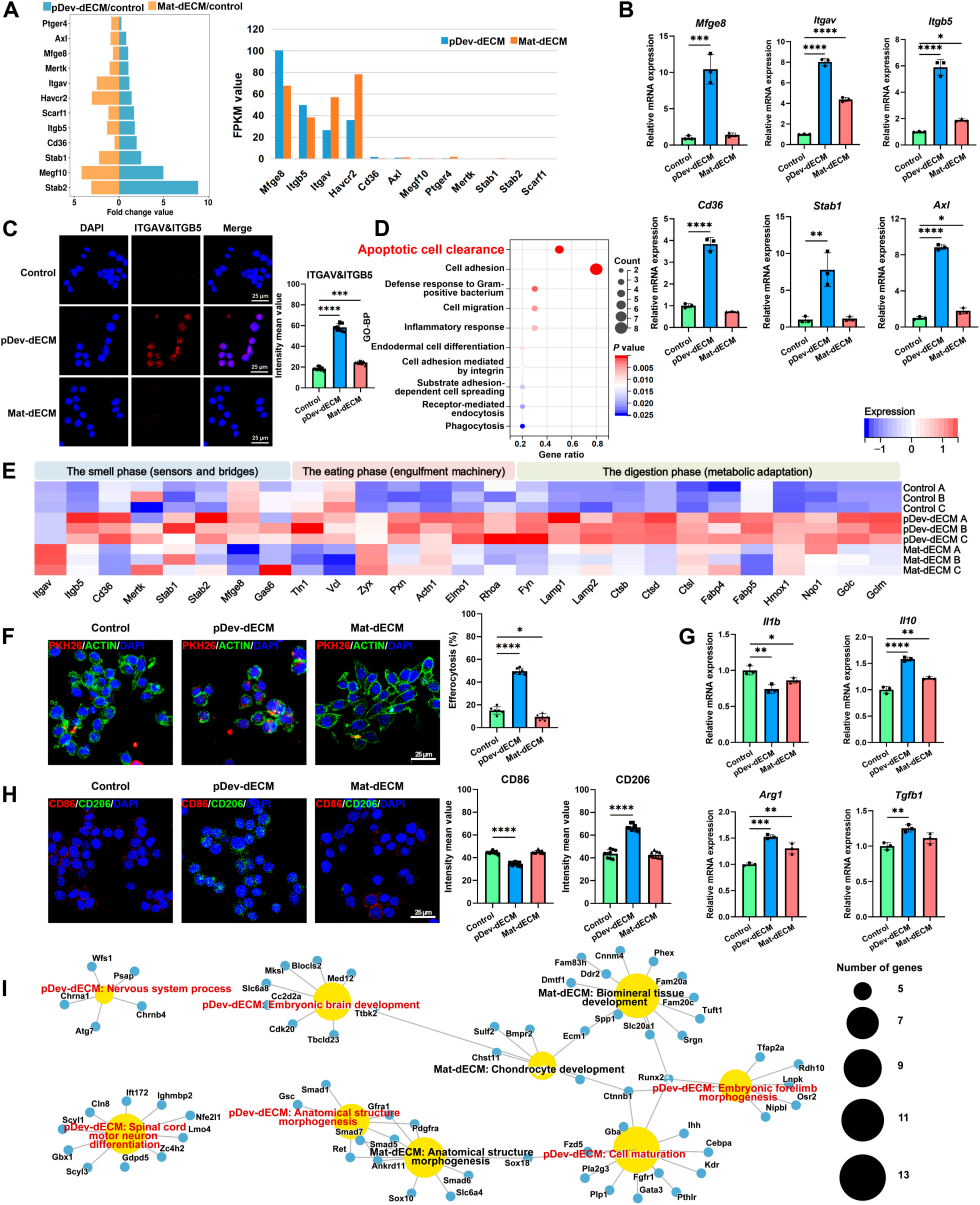
Transcriptomic and functional analysis reveals pDev-dECM-mediated macrophage reprogramming and immune microenvironment remodeling. (A) Fold change and fragments per kilobase per million (FPKM) values of adhesion- and recognition-related genes in pDev-dECM and Mat-dECM groups. (B) Reverse transcription quantitative polymerase chain reaction (RT-qPCR) validation of adhesion- and recognition-related gene expression (*n* = 3 per group). (C) Representative immunofluorescence images and quantification of ITGAV&ITGB5 expression in adult macrophages (*n* = 9 per group). (D) GO-BP analysis of highly expressed genes associated with adhesion and recognition in the pDev-dECM group. (E) Heatmap of genes mapped to the sequential stages of efferocytosis. (F) Representative fluorescent images of macrophages (stained with phalloidin–fluorescein isothiocyanate, green) cocultured with apoptotic periodontal ligament cells (PDLCs) (labeled with PKH26, red). Efferocytosis efficiency was calculated as the percentage of macrophages engulfing apoptotic PDLCs relative to total macrophages (*n* = 6 per group). (G) RT-qPCR analysis of pro- and anti-inflammatory gene expression in macrophages following efferocytosis (*n* = 3 per group). (H) Representative immunofluorescence images and quantification of CD86 and CD206 expression in macrophages following efferocytosis (*n* = 9 per group). (I) GO-BP network diagram illustrating tissue and organ development-related events in pDev-dECM and Mat-dECM groups. Data were presented as means ± SD. Significance was determined by one-way ANOVA with Tukey’s post hoc test. *P < 0.05, **P < 0.01, ***P < 0.001, and ****P < 0.0001.

Focusing on the adhesion and recognition machinery, gene analysis (Fig. 3A) showed that, while *Itgav* transcripts were abundant in Mat-dECM, pDev-dECM uniquely coordinated *Itgav* with *Itgb5*, as confirmed by robust protein-level assembly of the functional integrin αvβ5 heterodimer (Fig. 3B and C). Crucially, milk fat globule-epidermal growth factor-factor 8 (*Mfge8*), the bridging ligand connecting phosphatidylserine on apoptotic cells to αvβ5 receptors [40,41], was exclusively up-regulated in pDev-dECM (Fig. 3B). GO-BP enrichment linked these to apoptotic cell clearance and cell migration (Fig. 3D), supported by an integrated proteomic–transcriptomic–regulatory network (Fig. S3D).

Deeply dissecting the molecular drivers via the smell–eat–digest efferocytosis lifecycle map (Fig. 3E), we found that pDev-dECM induces a hypersensitized smell state via scavengers (*Cd36* and *Mertk*) and bridging molecules (*Gas6*). The eat machinery was structurally primed via adaptors (*Tln1* and *Pxn*) and drivers (*Elmo1* and *Rhoa*). Furthermore, a protective digest program was initiated. Gene Set Enrichment Analysis (GSEA) confirmed *Fyn*-orchestrated *Nrf2* activation (Fig. S3E) inducing antioxidant (*Hmox1* and *Gclc*) and lipid regulators (*Fabp4*). Thus, pDev-dECM primes a sensing-ready, metabolically adapted state. Functional assays (Fig. 3F) confirmed this outcome: pDev-dECM substantially enhanced apoptotic cell clearance via the phosphatidylserine–Mfge8–integrin αvβ5 axis, whereas Mat-dECM failed to promote phagocytosis.

Postinternalization, macrophages undergo metabolic reprogramming that dictates their inflammatory state [34,42,43]. With apoptotic periodontal ligament cells (PDLCs), reverse transcription quantitative polymerase chain reaction (RT-qPCR) (Fig. 3G) showed that pDev-dECM significantly down-regulated proinflammatory *Il1β* while up-regulating anti-inflammatory genes *(Il10*, *Tgfβ1*, and *Arg1)*. Immunofluorescence (Fig. 3H) corroborated this shift, displaying reduced CD86 (M1) and increased CD206 (M2) expression. These results indicated effective reprogramming into an anti-inflammatory M2 phenotype resembling the in vivo Macro_Dev subset.

Exploring contributions to hierarchical regeneration, enrichment analysis (Fig. 3I) revealed functional divergence: Mat-dECM favored hard tissue development, whereas pDev-dECM activated broader pathways related to nervous system development, limb morphogenesis, and cell maturation. This highlights pDev-dECM’s capacity for cross-organ morphogenesis versus Mat-dECM’s osteogenic specificity. Key genes driving these pathways included developmental signaling regulators (*Ctnnb1*, *Smad1*, *Smad5*, and *Ihh*) [44–48] and organogenesis-related transcription factors (*Runx2*, *Gata3*, and *Tfap2a*) [49–51]. Collectively, pDev-dECM remodels the microenvironment by inducing macrophages to secrete pleiotropic signals, orchestrating hierarchical cross-organ regeneration.

### pDev-dECM composite drives hierarchical periodontal regeneration via macrophage integrin αvβ5-mediated efferocytosis

To address dECM limitations in degradation resistance and spatial retention, we integrated pDev-dECM with biological hydroxyapatite (BHA) scaffolds [52]. SEM showed a dECM-smoothed microporous surface (Fig. S4A), while FTIR verified stable integration, retaining BHA’s phosphate/carbonate peaks alongside dECM’s characteristic amide bands (Fig. S4B).

**Fig. 4.**
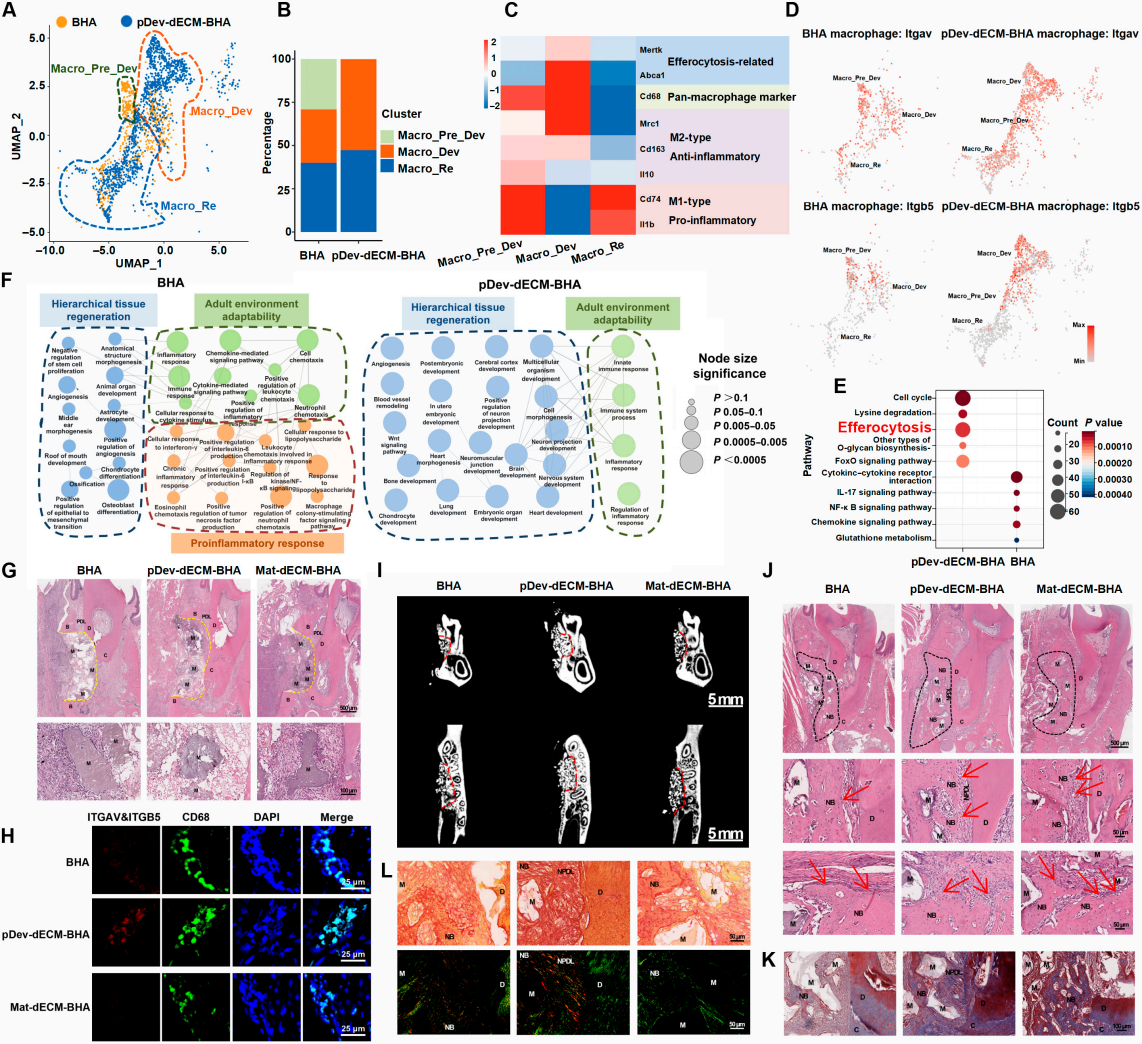
Single-cell and histological validation of pDev-dECM-mediated in situ developmental microenvironment recapitulation and periodontal hierarchical regeneration. (A) UMAP atlas of macrophage subsets in biological hydroxyapatite (BHA) and pDev-dECM-BHA groups. (B) Quantification of macrophage subset distribution. (C) Heatmap displaying the expression profiles of pan-macrophage, M1/M2 polarization, and efferocytosis-related markers across subsets. (D) Expression of *Itgav* and *Itgb5* genes in macrophage subsets in BHA and pDev-dECM-BHA groups. (E) KEGG enrichment analysis of DEGs between BHA and pDev-dECM-BHA groups. (F) Network diagram classifying enriched biological events related to adult environment adaptability and hierarchical organ regeneration. (G) H&E staining images of rat periodontal defect at day 7. (H) Representative immunofluorescence images of CD68 and ITGAV&ITGB5 of rat periodontal defects at day 7. (I and J) Microcomputed tomography reconstruction and H&E staining showing periodontal regeneration outcomes at 4 weeks postsurgery. (K) Masson’s trichrome staining assessing bone mineralization at 4 weeks. (L) Picrosirius red staining viewed under polarized light visualizing collagen regeneration and alignment in the defect area at 4 weeks. PDL, periodontal ligament; NPDL, newly formed periodontal ligament; B, alveolar bone; NB, newly formed alveolar bone; C, cementum; D, dentin; M, materials.

Dissecting in vivo immune regulation via scRNA-seq at day 7 (Fig. S5A), quantitative analysis revealed dramatic remodeling (Fig. 4A and B). The BHA group was dominated by proinflammatory Macro_Re, with the remaining fraction distributed between Macro_Pre_Dev and Macro_Dev. Crucially, while Macro_Pre_Dev in the BHA group showed acute surface activation (*Cd163* and *Il10*), they lacked functional competence. Conversely, pDev-dECM-BHA drove a decisive shift toward Macro_Dev, uniquely distinguished by the specific enrichment of the efferocytosis execution machinery (*Mertk* and *Abca1*) required for regeneration (Fig. 4C). While dot plot analysis (Fig. S5B) confirmed *Itgb5* expression in Macro_Dev across groups, spatial analysis (Fig. 4D) demonstrated that pDev-dECM substantially expanded the absolute abundance of this functional population compared to the sparse BHA control, ensuring robust tissue-level efferocytosis machinery. KEGG enrichment (Fig. 4E) confirmed that these macrophages were enriched in efferocytosis and cell cycle pathways. Functional enrichment analysis further elucidated the specific biological roles of macrophage subpopulations (Fig. 4F). The pDev-dECM-BHA group exhibited broad enrichment in biological processes critical for hierarchical organ regeneration (e.g., tissue morphogenesis and regulation of cell differentiation), whereas the BHA group remained locked in proinflammatory events (e.g., inflammatory response). This confirms pDev-dECM-BHA’s ability to suppress inflammation and adapt to the adult environment while specifically promoting regenerative functions.

We next investigated how this immune microenvironment influences mesenchymal cell behavior. GO-BP enrichment (Fig. S5C) revealed distinct modes: BHA mesenchymal cells favored terminal cell-mediated tissue repair (e.g., bone remodeling), whereas pDev-dECM-BHA induced stem-cell-mediated mesenchymal condensation, characterized by ECM deposition, morphogenetic induction, and mechanical compaction [14]. KEGG analysis (Fig. S5D) further distinguished the groups, showing cytoskeletal regulation and ECM–receptor enrichment in pDev-dECM-BHA versus mitogen-activated protein kinase/inflammatory signaling in BHA. Thus, pDev-dECM reprograms the niche from inflammatory repair to developmental reconstruction via macrophage–mesenchymal cross-talk.

Histopathological analysis at day 7 (Fig. 4G) and immunofluorescence (Fig. 4H and Fig. S5E) confirmed the specific recruitment of ITGAV^+^/ITGB5^+^ macrophages in the pDev-dECM-BHA group. Multiplex immunofluorescence (Fig. S5F) provided high-resolution phenotypic mapping. Consistent with scRNA-seq, pDev-dECM-BHA was dominated by CD68^+^CD206^+^ M2-like macrophages, while BHA retained high CD68^+^CD86^+^ M1-like macrophages (Fig. S5G). Quantitative cell counting confirmed a significant in situ shift toward M2 populations. Furthermore, enhanced CD10^+^ stem cells recruitment and fibronectin 1 (FN1) reaggregation (Fig. S5H) in pDev-dECM-BHA linked this immune modulation to mesenchymal activation.

Hierarchical regeneration was assessed at 4 weeks. Microcomputed tomography (Fig. 4I and Fig. S5I) revealed superior bone volume in the pDev-dECM-BHA group. Histologically (Fig. 4J and K), the control group showed limited bone formation and fibrous encapsulation, whereas pDev-dECM-BHA achieved extensive alveolar bone regeneration and physiological contour restoration. Notably, only the pDev-dECM-BHA group exhibited functionally oriented periodontal fibers perpendicular to the tooth surface (Fig. 4L), confirmed by dense yellow–red birefringence and the highest type I/III collagen ratio (Fig. S5J) [53,54]. In conclusion, pDev-dECM-BHA recapitulates the developmental macrophage–M2 polarization–mesenchymal condensation cascade, driving orderly, hierarchical regeneration.

### pDev-dECM promotes hierarchical cross-organ regeneration

**Fig. 5.**
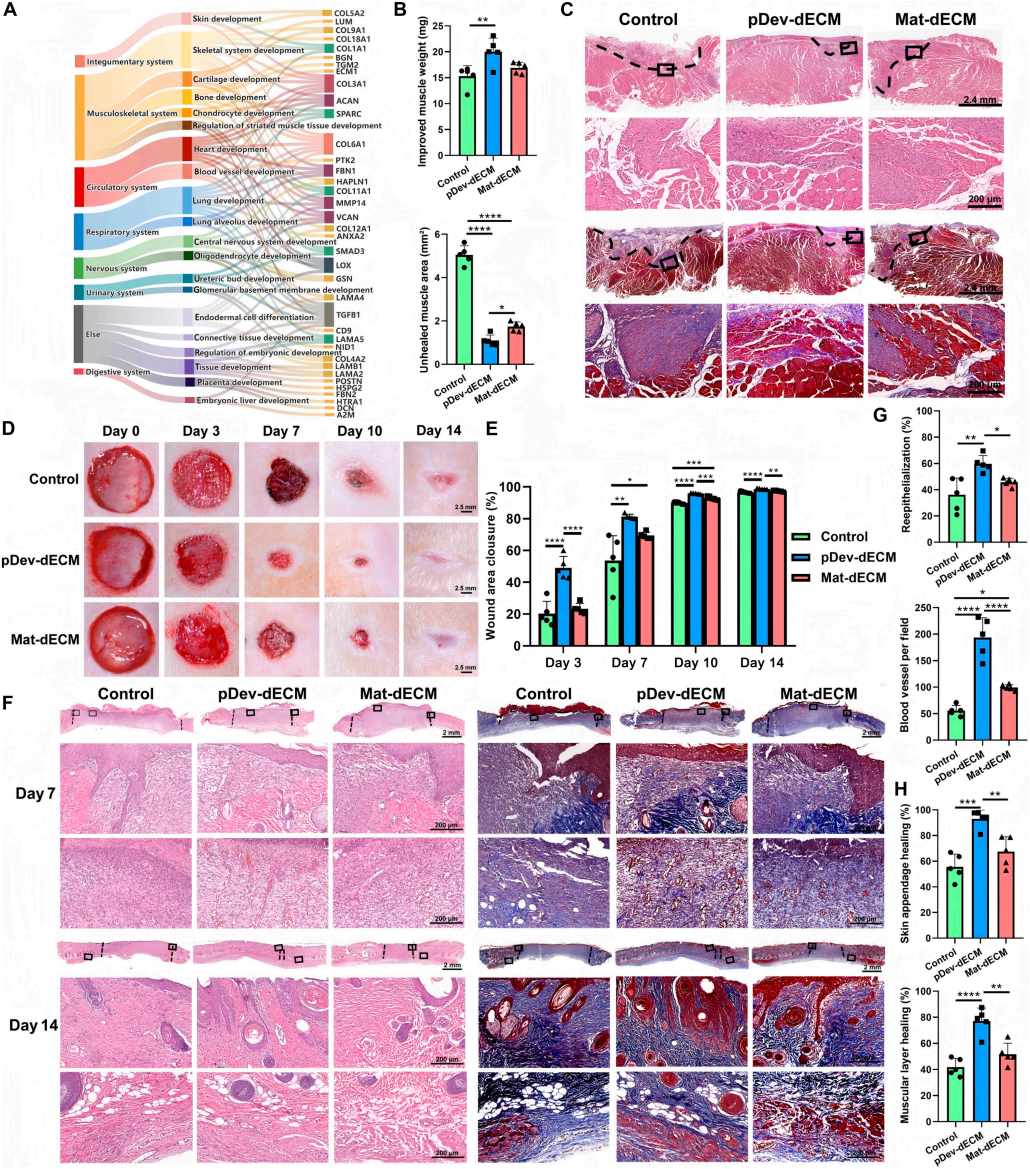
pDev-dECM promotes hierarchical cross-organ regeneration in skin and muscle models. (A) Sankey plot mapping ECM-associated proteins in the pDev-dECM proteome to developmental processes across multiple systems. (B) Quantification of tibialis anterior (TA) muscle weight recovery and percentage of unhealed muscle area in the volumetric muscle loss (VML) model at 4 weeks (*n* = 5 per group). (C) H&E and Masson’s trichrome staining of regenerated muscle at the VML site at 4 weeks. (D and E) Representative photographs and quantification of skin wound closure at days 3, 7, 10, and 14 (*n* = 5 per group). (F) H&E and Masson’s trichrome staining of skin defects at days 7 and 14. (G) Quantification of reepithelialization rate and blood vessel number at day 7 (*n* = 5 per group). (H) Quantification of skin appendage regeneration and muscle layer healing at day 14 (*n* = 5 per group). Data were presented as means ± SD. Significance was determined by one-way ANOVA with Tukey’s post hoc test. *P < 0.05, **P < 0.01, ***P < 0.001, and ****P < 0.0001.

Proteomic (Fig. 5A) revealed pDev-dECM enrichment in proteins associated with the development of multiple systems, including epidermal, musculoskeletal, circulatory, respiratory, and nervous systems. Hypothesizing that pDev-dECM could transcend its odontogenic origin to facilitate cross-organ regeneration (based on findings in Fig. 3I), we selected skeletal muscle (representing mesodermal tissue) and skin (representing ectodermal tissue) as distinct, clinically relevant models to rigorously validate the universal bioactivity of the pDev-dECM platform. To address volumetric muscle loss (VML) volume challenges, we delivered dECM via an inert gelatin sponge, whereas direct hydrogel was used for skin defects. Efficacy was compared against Mat-dECM and respective negative controls (phosphate-buffered saline [PBS]-loaded sponge for muscle; untreated for skin).

In the tibialis anterior (TA) VML model, regeneration was assessed at 4 weeks. While large defects typically result in fibrosis [55,56], pDev-dECM yielded substantially greater muscle weight recovery than PBS-loaded sponge controls (Fig. 5B). Histological analysis (Fig. 5C) revealed a qualitative divergence: Controls were filled with the sponge framework and extensive fibrosis, whereas pDev-dECM-treated defects achieved near-complete healing with organized fiber bundles resembling native tissue. Notably, while pDev-dECM and Mat-dECM showed comparable muscle weight gain, histopathology confirmed superior structural restoration in the pDev-dECM group, contrasting with the incomplete myogenesis and residual fibrosis in the Mat-dECM group. Thus, pDev-dECM drives functional regeneration rather than scaffold-mediated fibrotic filling.

In a 15-mm full-thickness skin defect model, pDev-dECM accelerated healing at all time points (Fig. 5D and E). Histologically (Fig. 5F), pDev-dECM enhanced reepithelialization and neovascularization as early as day 7 (Fig. 5G). By day 14, although all groups achieved >90% closure, tissue quality differed substantially (Fig. 5F and H), pDev-dECM restored hierarchical layers, including functional appendages and the panniculus carnosus [57,58]. Conversely, controls retained unhealed granulation tissue, with Mat-dECM showing intermediate outcomes.

Collectively, these findings confirm that pDev-dECM possesses intrinsic cross-tissue regenerative bioactivity, effectively promoting hierarchical repair in both muscle (mesoderm) and skin (ectoderm) tissues beyond its odontogenic origin.

## Discussion

**Fig. 6.**
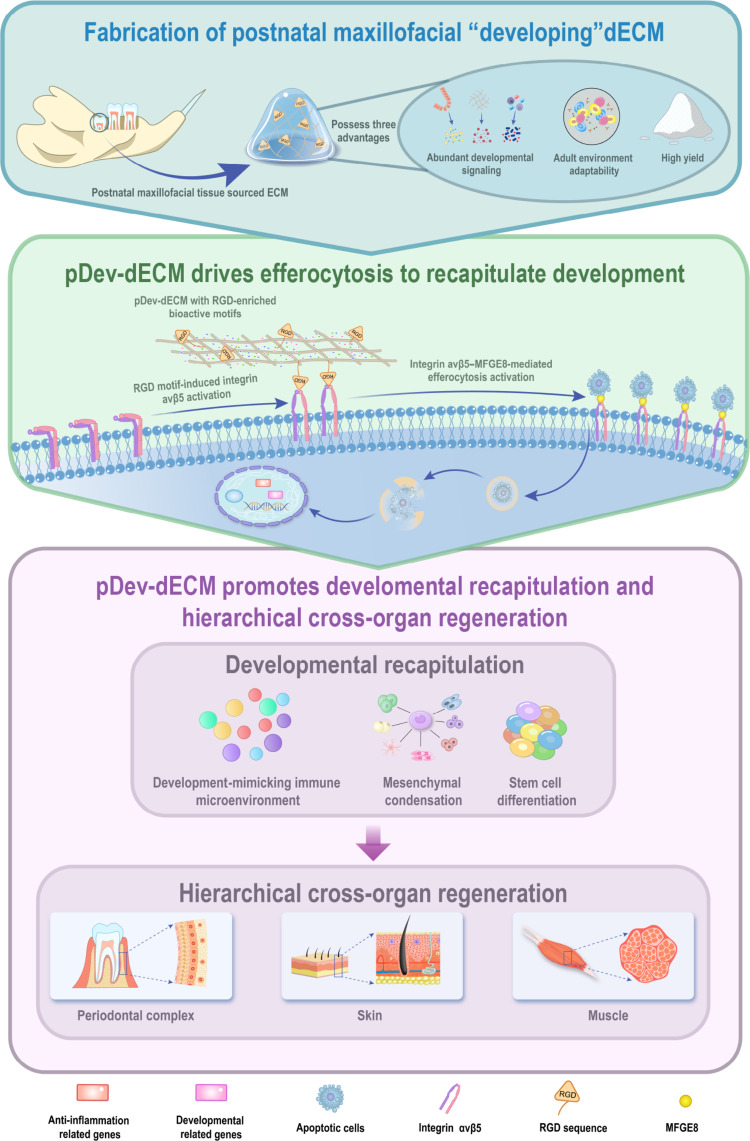
Schematic illustration of the pDev-dECM-mediated developmental reinitiation mechanism. pDev-dECM retains developmentally critical RGD-enriched bioactive motifs (validated proteomically in Fig. 1H), which prime macrophage reprogramming via the milk fat globule-epidermal growth factor-factor 8 (Mfge8)–integrin αvβ5 axis. Engagement of this axis triggers downstream cytoskeletal remodeling signals (molecular mechanism detailed in Fig. 2G), driving actin-dependent efferocytosis (functionally verified in Fig. 3E), and polarization toward regenerative phenotypes (quantified in Fig. 4B). Subsequent transcriptomic reprogramming activates anti-inflammatory and developmental gene programs (functional enrichment in Fig. 4F), which coordinate with mesenchymal stem cells to recapitulate embryonic processes (mesenchymal redevelopment in Fig. S5C). Collectively, this mechanism enables the hierarchical cross-organ regeneration of periodontal, skin, and muscle tissues (histologically confirmed in Figs. 4 and 5).

Our study established that pDev-dECM leveraged trans-stage developmental cues to mediate adult developmental recapitulation (Fig. 6), proposing it as a universal platform unlike traditional tissue-specific dECM. By preserving RGD-enriched bioactive components, pDev-dECM activated the Mfge8–integrin αvβ5 axis. This mechanism polarizes macrophages toward an M2 phenotype while orchestrating hierarchical regeneration across anatomical boundaries. Although we classified Macro_Dev as M2-like based on canonical markers, we acknowledge that macrophage polarization in vivo exists on a continuous spectrum. Therefore, Macro_Dev likely represents a specialized phenotype functionally adapted to the developmental niche, exhibiting a distinct transcriptomic signature that extends beyond the classic M2 definition.

Mechanistically, regenerative potency relies on a priming–execution paradigm. While in vitro data confirmed pDev-dECM primes macrophages by up-regulating recognition receptors (*Itgav*, *Itgb5*, and *Mfge8*), in vivo profiling (Fig. 2G) revealed that efferocytosis execution is triggered within the defect microenvironment. Specifically, the Macro_Dev subset activated the *Ptk2*–*Crk*–*Dock1* signaling cascade and cytoskeletal remodeling genes, driving physical engulfment. This mirrors conserved regenerative paradigms seen in cardiac and hepatic repair [34,59].

Following immunomodulation, pDev-dECM reprograms the niche by up-regulating developmental regulators (*β-catenin* and *Smad1/5*, *Ihh*) and lineage factors (*Runx2* and *Gata3*). This establishes a pseudo-developmental niche via reinitiated embryonic matrix deposition and morphogen gradients. Such reprogramming overrides adult fibrotic constraints, enabling the observed hierarchical regeneration.

Critically, this study challenges dECM tissue specificity limits. We utilized the periodontal complex, requiring synchronized hard/soft tissue regeneration, as a rigorous stress test. Successful hierarchical regeneration in this complex environment validates pDev-dECM’s robustness, explaining its efficacy in structurally simpler ectodermal (skin) and mesodermal (muscle) tissues. Thus, pDev-dECM transcends its maxillofacial origin as a broad-spectrum therapeutic.

Translationally, pDev-dECM represents a paradigm shift from traditional structural replacement to developmental reprogramming. Our optimized protocol achieved superior yields compared to mature tissues (Fig. 1B), likely due to the intrinsic lower cross-linking density of developing ECM, which facilitates efficient decellularization and solubilization. Ethically, unlike embryonic tissues that face stringent regulatory hurdles, pDev-dECM can be sustainably sourced from medical waste in dental clinics, such as extracted primary teeth, impacted wisdom teeth, or orthodontic extractions in diphyodont mammals [25–27]. This readily available source supports scalable manufacturing. To ensure clinical safety and efficacy, strict quality control measures must be implemented to manage batch-to-batch consistency. These include rigorous quantification of residual DNA (<50 ng/mg) to prevent immunogenicity, standardization of total collagen/GAG content, and rheological testing to ensure consistent injectability [30]. Future strategies could further exploit natural developmental windows, harvesting phase-specific biomaterials from reservoirs such as alveolar septation (lung) [60], synaptic pruning (neural) [61,62], or steroidogenic maturation (gonadal) [63,64] phases.

Cross-organ regeneration remains challenging for complex structures such as the esophagus and trachea [65,66], where current heterogeneous scaffolds lack developmental bioinductivity [67,68]. Our findings suggest that phase-specific harvesting can reactivate developmental plasticity universally. Future translational efforts should focus on establishing good-manufacturing-practice-compliant production lines, validating patterning accuracy in endodermal organ systems, and integrating hybrid scaffolds (e.g., 3-dimensional printing) with matrix bioinductivity to meet patient-specific anatomical needs.

While our findings highlight the transformative potential of pDev-dECM, several limitations warrant mention. First, our mechanistic conclusions regarding the Mfge8–integrin αvβ5 axis rely primarily on transcriptomic signatures, proteomic correlation, and comparative phenotypic assays. Although the specific up-regulation of this pathway in the pDev-dECM group strongly correlates with functional efferocytosis, definitive causality and direct visualization of the dynamic cascade were not established via genetic ablation or live imaging. Future studies utilizing *Mfge8*^−/−^ or *Itgav*-floxed models will be necessary to strictly isolate the contribution of this molecular axis. Second, although we demonstrated cross-organ efficacy in skin and muscle models, the specific cellular interactomes in these nondental tissues remain to be fully characterized at single-cell resolution.

## Conclusion

By leveraging the trans-stage ontogeny of maxillofacial odontogenic tissues, we developed pDev-dECM, a developmentally dynamic matrix that reinitiates adult developmental potential through embryonic niche reactivation. This material synergizes intrinsic root-periodontal morphogenetic cues with adult microenvironment adaptability, driving hierarchical regeneration of periodontal, cutaneous, and muscular tissues by inducing integrin αvβ5-mediated efferocytosis. We propose a developmental phase-locked strategy, where timed ECM extraction captures critical morphogenetic signals. Ultimately, our work redefines regenerative biomaterials, demonstrating that phase-specific dECM transcends tissue-specific limitations to execute developmental blueprints, offering a universal platform for hierarchical cross-organ regeneration through evolutionarily conserved ontogenetic pathways.

## Materials and Methods

### Preparation of pDev-dECM and Mat-dECM

The animal experimental protocols were approved by the Institutional Animal Care and Use Committee of Sun Yat-sen University (SYSU-IACUC-2023-001038). Male Sprague–Dawley rats aged 20 d (pDev-dECM group) and 3 months (Mat-dECM group) were selected. Mandibles were harvested and decalcified in 10% ethylenediaminetetraacetic acid (EDTA; Biosharp, China) solution for 4 weeks. The decalcified tissues were decellularized in 1% Triton X-100 (Solarbio, China) and 1% sodium dodecyl sulfate (Biosharp, China) solution for 24 h, respectively, followed by mechanical mincing. The resulting fragments were digested with pepsin (1 mg/ml; Sigma-Aldrich, Germany) solution containing 0.01 M hydrochloric acid (Aladdin, China) for 48 h. Following digestion, sodium hydroxide (Aladdin, China) was added to adjust the pH to 7.4 for pepsin inactivation. The supernatant was lyophilized for 24 h to obtain dECM powder. To quantify the extraction efficiency, the dECM yield was calculated as the ratio of the final lyophilized dECM dry weight to the initial wet weight of the mandibular tissue prior to decalcification. Finally, the powder was reconstituted in sterile PBS at a concentration of 100 mg/ml, and the solution was incubated at 37 °C for 1 h to induce hydrogel formation.

### Characterization of pDev-dECM and Mat-dECM

To evaluate decellularization efficacy, native and decellularized mandibles were fixed with 4% paraformaldehyde for 24 h. H&E (Servicebio, China) and DAPI (Beyotime, China) staining were performed. Images were captured using an Aperio AT2 system (Leica, Germany) and a fluorescence microscope (Zeiss, Germany), respectively. For DNA quantification, genomic DNA was extracted from native and decellularized tissues using the Universal Genomic DNA Kit (CWBIO, China) and quantified using a NanoDrop 2000 spectrophotometer (Thermo Fisher Scientific, USA). The surface micromorphology of hydrogels was characterized by SEM (JEOL, Japan). FTIR spectra were acquired using an FTIR microscope (Thermo Fisher Scientific, USA) in the wavelength range of 400 to 4,000 cm^−1^.

### Comparative proteomic analysis of pDev-dECM and Mat-dECM

Triplicate samples of lyophilized pDev-dECM and Mat-dECM were prepared for proteomic analysis. After protein extraction, enzymatic digestion, and high pH reversed-phase separation, data-dependent acquisition and data-independent acquisition analyses were performed via nano-liquid chromatography–tandem mass spectrometry. The data-independent acquisition data were analyzed using indexed retention time peptides for retention time calibration. To ensure quantitative reliability, a target–decoy model applicable to sequential window acquisition of all theoretical mass spectra was applied with a false discovery rate set at 1%. Matrisome annotations were classified according to MatrisomeDB (https://matrisomedb.org/) [69,70]. GO and KEGG enrichment analyses were performed. All data visualization was conducted using GraphPad Prism (v 8.0.1), Cytoscape (v 3.9.1), Sangerbox (https://vip.sangerbox.com), and OmicStudio tools (https://www.omicstudio.cn/tool).

### Cell isolation, culture, and apoptosis induction

Human PDLCs were isolated from healthy teeth of subjects aged <25 years who required third molar extraction or orthodontic treatment. Subsequent experiments were conducted using PDLCs at passages 3 to 5. PDLCs were cultured in minimum essential medium (Gibco, USA), supplemented 10% fetal bovine serum (ExCell, China) and 1% penicillin/streptomycin (Gibco, USA). Cells were maintained in a humidified incubator at 37 °C with 5% CO_2_. For apoptosis induction, PDLCs were treated with minimum essential medium containing steroid sulfatase (500 nM; Enzo Life Sciences, USA) at 37 °C for 6 h. Apoptotic PDLCs were collected by centrifugation at 800*g* for 10 min.

The RAW 264.7 cell line was cultured in Dulbecco’s modified Eagle’s medium (Gibco, USA), supplemented with 10% fetal bovine serum (ExCell, China) and 1% penicillin/streptomycin (Gibco, USA). For dECM coating, pDev-dECM and Mat-dECM hydrogels were applied to culture flasks and allowed to air-dry. Subsequently, RAW 264.7 cells were seeded into the dECM-coated flasks. For the cytotoxicity assay, cell viability was assessed using a Cell Counting Kit-8 (Dojindo, Japan) after 24 and 48 h of culture.

### Transcriptome analysis and RT-qPCR

After culture with pDev-dECM or Mat-dECM for 24 h, total RNA was extracted from RAW 264.7 cells using TRIzol reagent (Beyotime, China). RNA-seq was performed using the BGISEQ-500 platform at BGI (Shenzhen, China). Raw data in FASTQ format were processed to calculate read counts and FPKM for further analysis. PCA, GO, and KEGG analyses were conducted using the Dr. Tom system (https://biosys.bgi.com). All data visualization was performed using GraphPad Prism (v 8.0.1), Sangerbox (https://vip.sangerbox.com), OmicStudio tools (https://www.omicstudio.cn/tool), and Metascape (https://metascape.org/gp/index.html).

For RT-qPCR, the concentration and purity of extracted total RNA were quantified using a NanoDrop spectrophotometer (Thermo Fisher Scientific, USA). RNA was then reverse-transcribed into complementary DNA (cDNA) using the Hifair II 1st Strand cDNA Synthesis SuperMix (Yeasen, China). Equivalent cDNA samples were analyzed by RT-qPCR analysis using the Hieff qPCR SYBR Green Master Mix (Yeasen, China) on an ABI 2-step system (Applied Biosystems, USA). Primer sequences are listed in the Supplementary Materials (Table S1).

### Immunofluorescence and detection of efferocytosis

Following 24 h of culture on pDev-dECM or Mat-dECM coatings, RAW 264.7 cells were fixed with 4% paraformaldehyde for 1 h. Cells were blocked with 1% bovine serum albumin (BSA; Macklin, China) solution for 1 h and incubated with antibody against ITGAV&ITGB5 (1:200; Santa Cruz Biotechnology, USA) overnight at 4 °C. Subsequently, cells were incubated with a goat anti-mouse secondary antibody (1:200; Beyotime, China) for 1 h. Nuclei were counterstained with DAPI as described above. Fluorescent images were acquired using a laser scanning confocal microscope (LSM980, Zeiss, Germany). The relative fluorescence intensities of ITGAV&ITGB5 were quantified using ImageJ software.

For the detection of efferocytosis, apoptotic PDLCs were labeled with PKH26 (Sigma-Aldrich, Germany) and then added to RAW 264.7 cells that had been cultured on dECM coatings for 24 h. The ratio of RAW 264.7 cells to apoptotic PDLCs was 1:5. Following cocultured for 3 h, cells were fixed with 4% paraformaldehyde for 1 h and stained with Actin-Tracker Green (Beyotime, China) and DAPI (Beyotime, China). Images were acquired using a laser scanning confocal microscope (LSM980, Zeiss, Germany).

To assess macrophage polarization after efferocytosis, RAW 264.7 cells were cocultured with apoptotic PDLCs for 3 h, fixed with 4% paraformaldehyde, and blocked with 1% BSA. Cells were incubated with anti-CD86 (1:1,000; Thermo Fisher Scientific, USA) and anti-CD206 (1:1,000, Abcam, UK) antibodies overnight at 4 °C, followed by incubation with a goat anti-rabbit secondary antibody (1:200; Beyotime, China) for 1 h. Nuclei were stained with DAPI. Images were acquired using a laser scanning confocal microscope, and relative fluorescence intensities were quantified using ImageJ software.

### Preparation and characterization of pDev-dECM-BHA and Mat-dECM-BHA

BHA was prepared according to previous studies [71–73]. Briefly, cancellous bone was harvested from the porcine femoral epiphyses and immersed in 30% H_2_O_2_ and anhydrous ethanol for 24 h to remove soft tissues. The bone was dissected into blocks and calcined in a muffle furnace (SGM6812BK, Sigma Furnace Industry, China) at 800 °C for 2 h. The calcined bone blocks were ground into particles and sieved to obtain BHA particles with a diameter of 0.2 to 0.5 mm. To prepare the composite, lyophilized pDev-dECM or Mat-dECM powder was reconstituted in sterile PBS, mixed with BHA particles, and incubated at 37 °C for 1 h. Finally, the composites were air-dried to obtain pDev-dECM-BHA and Mat-dECM-BHA. SEM (JEOL, Japan) and FTIR (Thermo Fisher Scientific, USA) analyses were performed as described above.

### Animal surgery and sampling

The animal experimental protocols were approved by the Institutional Animal Care and Use Committee of Sun Yat-sen University (SYSU-IACUC-2023-001018, SYSU-IACUC-2023-001038, and SYSU-IACUC-2025-001110).

#### In vivo regenerative efficacy evaluation

Male Sprague–Dawley rats aged 6 to 8 weeks were used to evaluate the regenerative efficacy of the materials.

For periodontal defect model, rats were randomly divided into 3 groups: BHA, pDev-dECM-BHA, and Mat-dECM-BHA. The experiment was performed according to our previous studies [74]. Briefly, a mandible defect (5 × 2 × 1 mm) was prepared on the buccal alveolar bone of the posterior tooth root area. Materials were implanted, and samples were collected at 7 and 28 d postsurgery for histological and radiographic analyses.

For the full-thickness skin defect model, defects with a diameter of 15 mm were created on the dorsal skin. Wounds were coated directly with dECM hydrogels (pDev-dECM and Mat-dECM) or left untreated. Wounds were photographed at 0, 3, 7, 10, and 14 d, and wound areas were accurately calculated using ImageJ (v 1.52a) software. Samples were collected at 7 and 14 d.

For the TA muscle VML model, rats were randomly divided into 4 groups: uninjured, PBS-loaded sponge control (control), pDev-dECM, and Mat-dECM. The VML injury model were established according the previous research [75], a muscle defect (7 × 10 × 3 mm) was excised. Considering the volumetric nature of VML defects, absorbable gelatin sponges (Yongning, China; 7 × 10 × 3 mm) were utilized as inert carriers for all implantation groups. Specifically, sponges were fully absorbed with pDev-dECM hydrogel, Mat-dECM hydrogel, or an equivalent volume of sterile PBS (control group) prior to implantation. This rigorous design ensured that the observed regenerative differences were attributable solely to the bioactive dECM components rather than the delivery vehicle. Samples were harvested at 28 d for muscle weight and histological analysis. The calculation method of muscle growth is referred to the relevant previous studies [56].

#### scRNA-seq sampling

Tissue samples were collected for scRNA-seq to construct developmental atlases and evaluate material responses. To map the transcriptomic landscape of natural periodontal ontogeny and physiological repair (Fig. 2), periodontal tissues were dissected from C57BL/6 mice at postnatal days 7 and 14 and 5 weeks (maturation), as well as from a murine physiological repair model (2 × 1 × 0.5 mm defect) at 2 and 4 weeks postsurgery [76,77]. To evaluate the specific immune microenvironment modulated by the composite materials (Fig. 4), tissues were harvested from the rat periodontal defect model (pDev-dECM-BHA and BHA groups) described above at 7 d postimplantation. All harvested tissues were preserved in MACS Tissue Storage Solution (Miltenyi Biotec) until processing.

### Histological analysis

Tissue samples (mandible, skin, and muscle) were fixed with 4% paraformaldehyde for 24 h. Mandible samples were subsequently decalcified in 10% EDTA solution for 4 weeks. H&E staining was performed to observe general morphology. For immunofluorescence staining, samples were blocked with 5% BSA (Macklin, China) solution for 1 h and incubated with ITGAV&ITGB5 (1:200; Santa Cruz Biotechnology, USA) and CD68 (1:100; Invitrogen, USA) overnight at 4 °C. Sections were then incubated with goat anti-rabbit and goat anti-rat secondary antibodies (1:200; Beyotime, China), followed by nuclear counterstaining with DAPI.

For multiplex immunofluorescence staining, antigen retrieval was performed, followed by blocking with 5% BSA for 1 h. Samples were incubated with primary antibodies against CD68 (1:100; Invitrogen, USA), CD206 (1:500; Abcam, UK), and CD86 (1:100; Thermo Fisher Scientific, USA) overnight at 4 °C. Sections were then incubated with goat anti-rabbit and goat anti-rat secondary antibodies (1:200; Beyotime, China), followed by nuclear counterstaining with DAPI.

For immunohistochemical staining, antigen retrieval was performed, followed by blocking with 5% BSA for 1 h. Samples were incubated with primary antibodies against FN1 (1:2,000; Abcam, UK) and CD10 (1:500; Abcam, UK) overnight at 4 °C. Subsequently, sections were incubated with the corresponding secondary antibody, developed with diaminobenzidine solution (Genetech, China), and counterstained with hematoxylin. Collagen deposition was assessed using Picrosirius Red (Servicebio, China) and Masson’s Trichrome staining (Solarbio, China). Images were acquired using an Aperio AT2 system and a laser scanning confocal microscope. Picrosirius-Red-stained samples were imaged using a polarized microscope (MF43, Mshot, China). Semiquantification was conducted using ImageJ software and ImageScope software.

### Single-cell RNA sequencing

scRNA-seq experiments were conducted by NovelBio Co. Ltd. Libraries were generated using the 10x Genomics Chromium Controller Instrument and Chromium Single Cell 3′ V3 Reagent Kits (10x Genomics, Pleasanton, CA). Raw sequencing data were aligned to the appropriate reference genomes and processed with Cell Ranger (v 7.1.0) software to generate single-cell gene expression matrices. Matrices were imported into the Seurat package (v 4.3.0) for downstream analysis. Quality control parameters were tailored to dataset characteristics. For the developmental atlas (encompassing development, maturation, and repair phases; Fig. 2), cells were filtered for mitochondrial genes of ≤10%, 300 to 10,000 detected genes, and 500 to 100,000 unique molecular identifiers. The regenerative periodontal defect model samples (Fig. 4) utilized adjusted thresholds (mitochondrial ≤ 20%; 200 to 8,000 genes) to preserve stress-responsive populations. Potential doublets were excluded via upper-limit cutoffs. The Seurat function FindVariableGenes was used to select variable features, followed by PCA and Uniform Manifold Approximation and Projection for dimensionality reduction and visualization. Cluster-enriched marker genes were identified using the FindAllMarkers function. The lists of the top 20 differentially expressed genes (ranked by avg_log2FC) and selected functional markers for macrophage subpopulations, included regardless of statistical thresholds to capture canonical identity, were provided in Tables S2 and S3. Visualization was performed using ggplot2 (v 4.3.0) for histograms, pheatmap (v 4.3.0) for heatmaps, and FeaturePlot for feature mapping. KEGG enrichment analysis was conducted using the clusterProfiler package (v 4.3.0) .

### Statistical methods

Each experiment was repeated at least 3 times independently, and the sample size (*n*) was presented in each figure legend. Data were presented as means ± standard deviations. Normality and homogeneity of variance were assessed using the Shapiro–Wilk test and Levene’s test, respectively. For comparisons between 2 groups, a 2-sided unpaired Student’s *t* test was used for data following a normal distribution; otherwise, the nonparametric Mann–Whitney *U* test was applied. Multigroup comparisons were analyzed by one-way analysis of variance (ANOVA), followed by Tukey’s post hoc test. All statistical analyses were performed using GraphPad Prism (v 8.0.1). *P* value < 0.05 was considered statistically significant (*P < 0.05, **P < 0.01, ***P < 0.001, and ****P < 0.0001).

## Data Availability

Data will be made available on request.
