# Supplementary material for: Postnatal Maxillofacial “Developing” Decellularized Extracellular Matrix Orchestrates Hierarchical Cross-Organ Regeneration via Macrophage Integrin αvβ5-Mediated Efferocytosis-Driven Developmental Recapitulation
Source: Research (Wash D C). 2026 Apr 15;9:1234. doi: 10.34133/research.1234 (PMC13080099; doi:10.34133/research.1234)
Supplement: Supplementary 1 — Figs. S1 to S5 Tables S1 to S3 [file research.1234.f1.zip › Supplementary Figure 5.pdf]

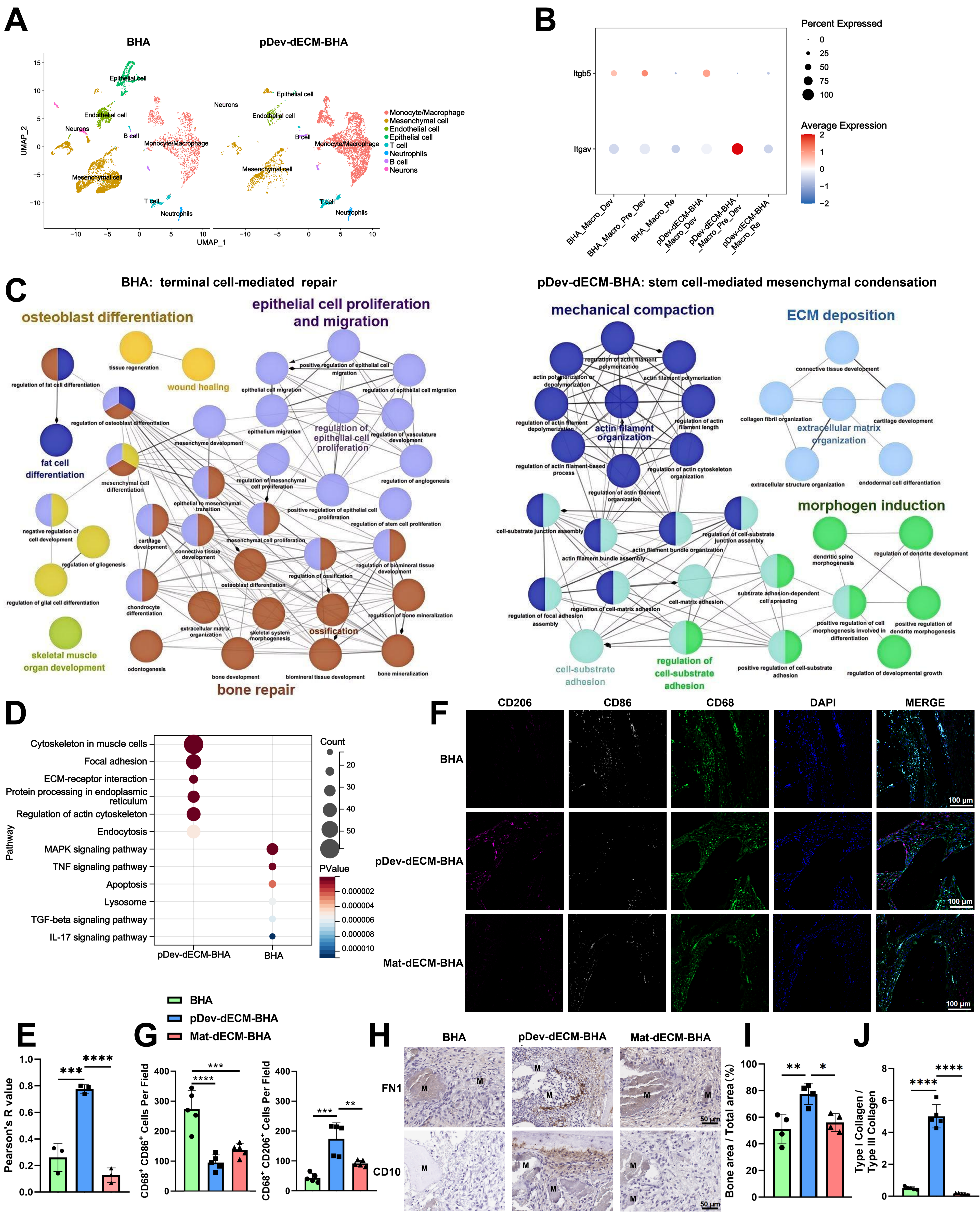

A
BHA
pDev-dECM-BHA
UMAP_2
UMAP_1
Epithelial cell
Endothelial cell
Neurons
B cell
Monocyte/Macrophage
Mesenchymal cell
T cell
Neutrophils
Monocyte/Macrophage
Mesenchymal cell
Endothelial cell
Epithelial cell
T cell
Neutrophils
B cell
Neurons
B
Percent Expressed
0
25
50
75
100
Average Expression
Itgb5
Itgav
BHA_Macro_Dev
BHA_Macro_Pre_Dev
BHA_Macro_Re
pDev-dECM-BHA _Macro_Dev
pDev-dECM-BHA _Macro_Pre_Dev
pDev-dECM-BHA _Macro_Re
C
BHA: terminal cell-mediated repair
osteoblast differentiation
epithelial cell proliferation and migration
fat cell differentiation
skeletal muscle organ development
bone repair
wound healing
tissue regeneration
regulation of fat cell differentiation
regulation of osteoblast differentiation
mesenchymal cell differentiation
negative regulation of cell development
regulation of gliogenesis
regulation of glial cell differentiation
cartilage development
connective tissue development
chondrocyte differentiation
odontogenesis
extracellular matrix organization
skeletal system morphogenesis
bone development
biomineral tissue development
bone mineralization
ossification
osteoblast differentiation
regulation of ossification
regulation of biomineral tissue development
regulation of bone mineralization
mesenchymal cell proliferation
epithelial to mesenchymal transition
regulation of mesenchymal cell proliferation
mesenchyme development
epithelium migration
epithelial cell migration
positive regulation of epithelial cell migration
regulation of epithelial cell migration
regulation of epithelial cell proliferation
regulation of vasculature development
regulation of angiogenesis
positive regulation of epithelial cell proliferation
regulation of stem cell proliferation
pDev-dECM-BHA: stem cell-mediated mesenchymal condensation
mechanical compaction
ECM deposition
morphogen induction
actin filament organization
extracellular matrix organization
regulation of actin filament polymerization
actin polymerization or depolymerization
actin filament polymerization
regulation of actin filament depolymerization
regulation of actin filament length
regulation of actin filament-based process
regulation of actin cytoskeleton organization
regulation of actin filament organization
cell-substrate junction assembly
regulation of cell-substrate junction assembly
actin filament bundle assembly
actin filament bundle organization
regulation of focal adhesion assembly
regulation of cell-matrix adhesion
cell-matrix adhesion
substrate adhesion-dependent cell spreading
cell-substrate adhesion
regulation of cell-substrate adhesion
positive regulation of cell-substrate adhesion
connective tissue development
collagen fibril organization
cartilage development
extracellular structure organization
endodermal cell differentiation
dendritic spine morphogenesis
regulation of dendrite development
positive regulation of cell morphogenesis involved in differentiation
positive regulation of dendrite morphogenesis
regulation of developmental growth
D
Pathway
Cytoskeleton in muscle cells
Focal adhesion
ECM-receptor interaction
Protein processing in endoplasmic reticulum
Regulation of actin cytoskeleton
Endocytosis
MAPK signaling pathway
TNF signaling pathway
Apoptosis
Lysosome
TGF-beta signaling pathway
IL-17 signaling pathway
pDev-dECM-BHA
BHA
Count
20
30
40
50
PValue
0.000002
0.000004
0.000006
0.000008
0.000010
F
CD206
CD86
CD68
DAPI
MERGE
BHA
pDev-dECM-BHA
Mat-dECM-BHA
100 μm
BHA
pDev-dECM-BHA
Mat-dECM-BHA
E
Pearson's R value
***
****
G
CD68⁺ CD86⁺ Cells Per Field
CD68⁺ CD206⁺ Cells Per Field
****
***
**
H
BHA
pDev-dECM-BHA
Mat-dECM-BHA
FN1
CD10
M
50 μm
I
Bone area / Total area (%)
**
*
J
Type I Collagen / Type III Collagen
****
****
