# Supplementary material for: Postnatal Maxillofacial “Developing” Decellularized Extracellular Matrix Orchestrates Hierarchical Cross-Organ Regeneration via Macrophage Integrin αvβ5-Mediated Efferocytosis-Driven Developmental Recapitulation
Source: Research (Wash D C). 2026 Apr 15;9:1234. doi: 10.34133/research.1234 (PMC13080099; doi:10.34133/research.1234)
Supplement: Supplementary 1 — Figs. S1 to S5 Tables S1 to S3 [file research.1234.f1.zip › Supplementary Material.docx]

**
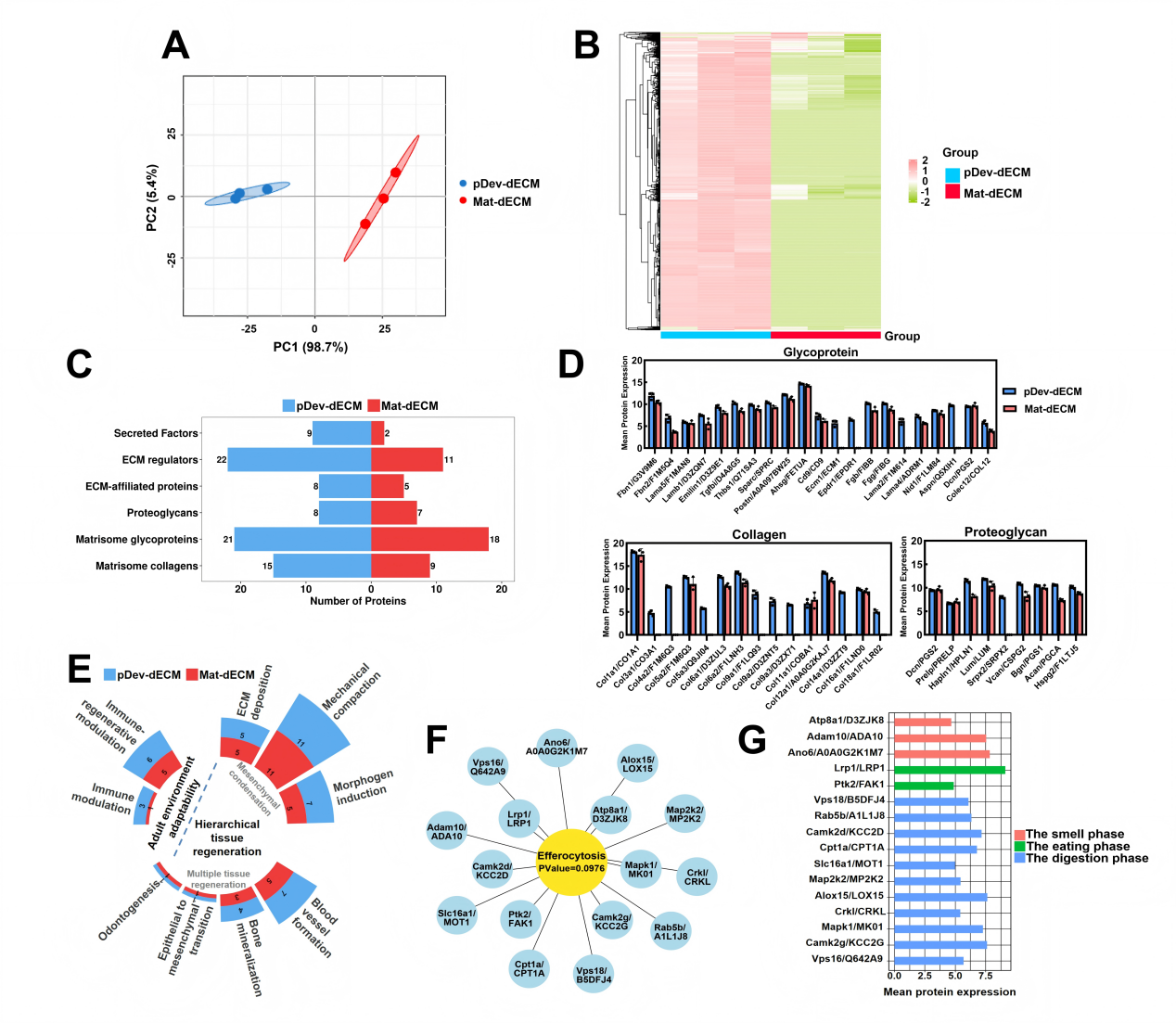
**

**Supplementary Fig. 1 Proteomic analysis of the pDev-dECM and Mat-dECM.** (A, B) PCA and overall protein expression heatmap of pDev-dECM and Mat-dECM (n=3 per group). (C) Matrisome classification of ECM proteins into five functional groups (n=3 per group). (D) Core matrisome expression levels in pDev-dECM vs. Mat-dECM (n=3 per group). (E) GO-BP enrichment analysis of ECM proteins ring bar chart of the number and classification of various enriched biological events of pDev-dECM and Mat-dECM. (F) Efferocytosis pathway network of pDev-dECM-specific proteins and their corresponding genes. (G) pDev-dECM specific efferocytosis pathway-related proteins and their expression were divided into three categories according to the stages of efferocytosis.


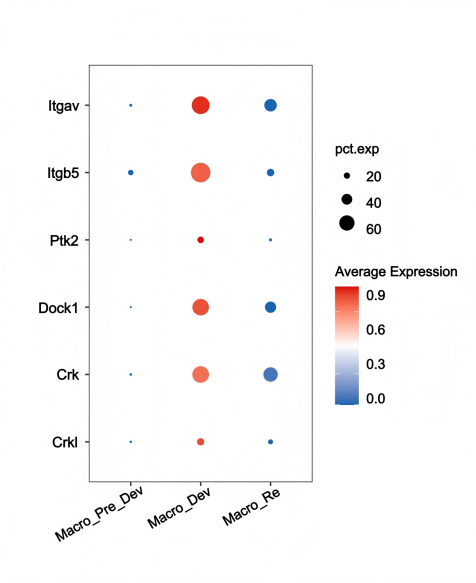


**Supplementary Fig. 2 Transcriptional co-expression of the integrin-mediated signaling cascade in macrophage subpopulations.**

**
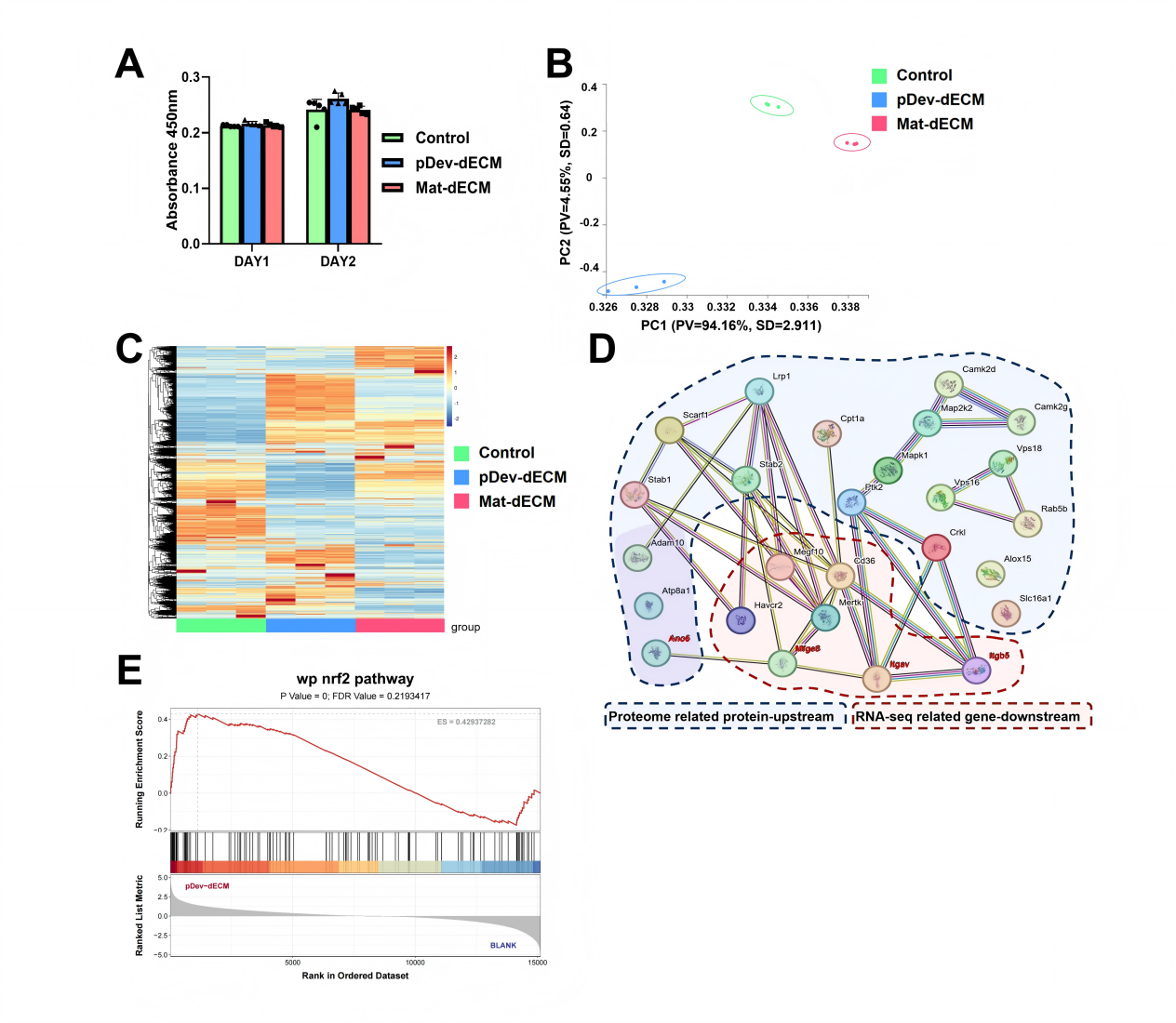
**

**Supplementary Fig. 3 Transcriptomic and cytotoxicity analysis of pDev-dECM, Mat-dECM, and control groups.** (A) Cytotoxicity assessment of pDev-dECM and Mat-dECM cocultured with RAW 264.7 using CCK-8 (n=5 per group). (B, C) PCA and overall gene expression heatmap of the three groups (n=3 per group). (D) The upstream and downstream network of the efferocytosis related proteins in proteome and the apoptotic cell clearance related genes in RNA-seq of pDev-dECM. (E) GSEA plot showing significant activation of the *Nrf2* Pathway (WP_NRF2_PATHWAY) in the pDev-dECM group. Data were presented as mean ± SD by one-way ANOVA with Tukey’s post hoc test. **P* < 0.05, ***P* < 0.01, ****P* < 0.001, *****P* < 0.0001.


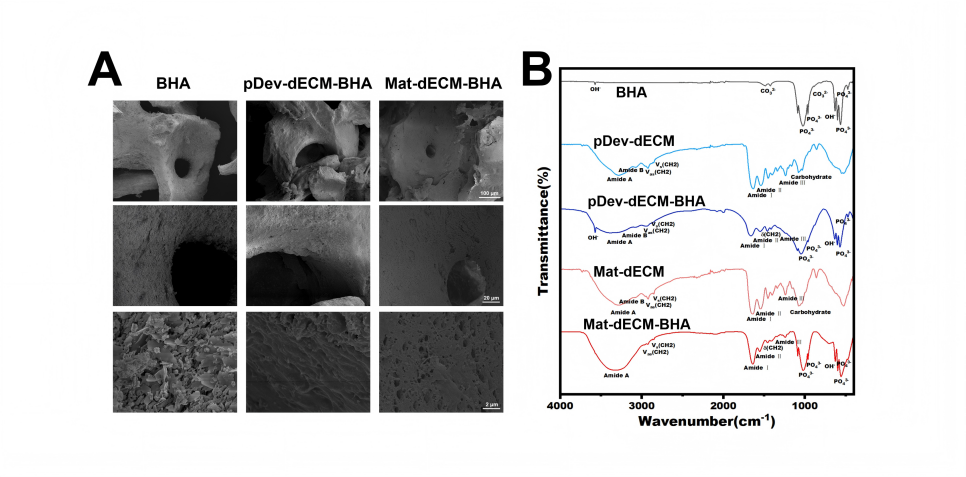


**Supplementary Fig. 4 Fabrication of dECM composite materials for hierarchical periodontal regeneration.** (A) Morphology and structural characterization of prepared BHA, pDev-dECM-BHA, and Mat-dECM-BHA detected by SEM. (B) FTIR analysis of BHA, pDev-dECM-BHA, and Mat-dECM-BHA.


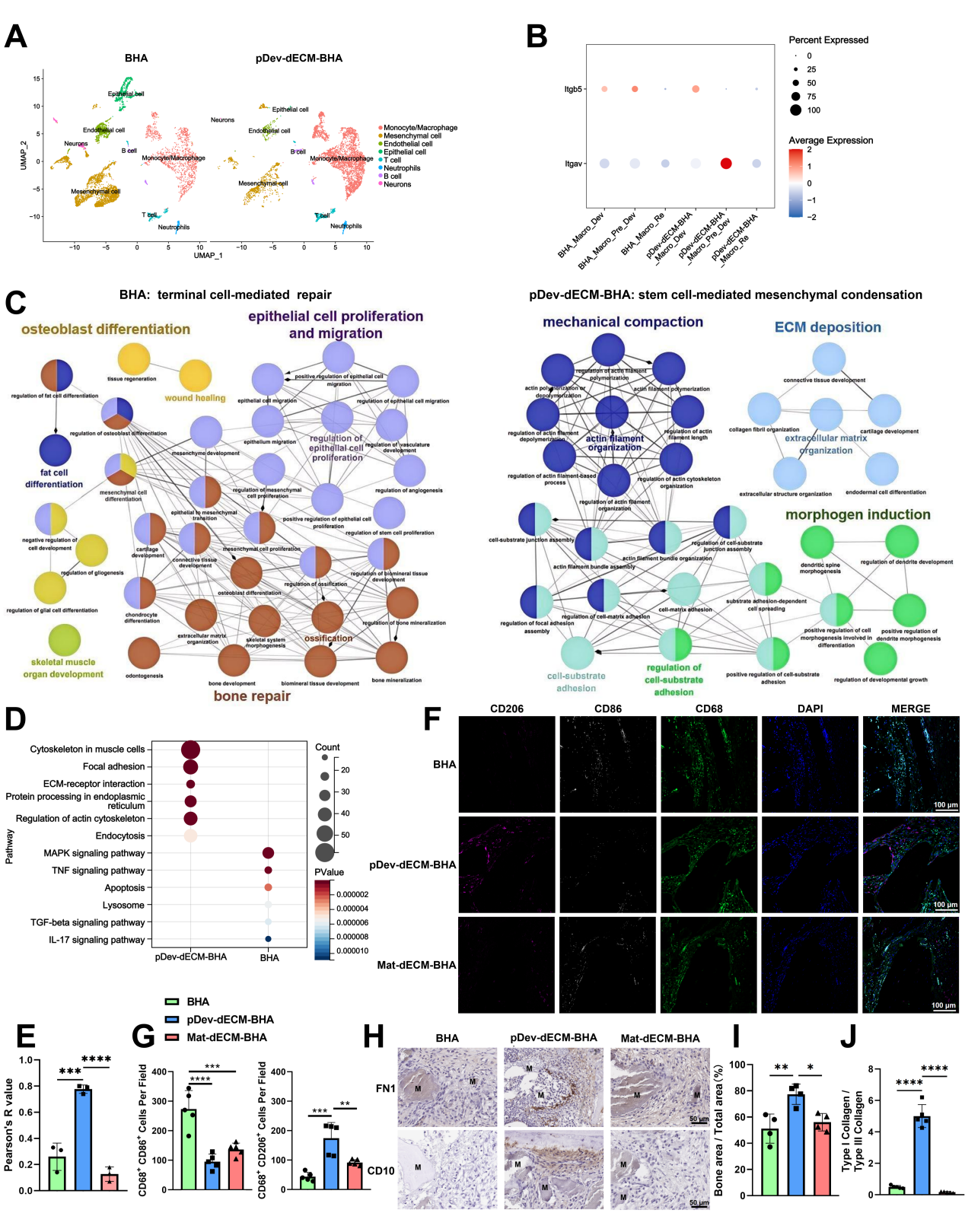


**Supplementary Fig. 5 Functional validation of dECM composite materials for hierarchical periodontal regeneration.** (A) UMAP atlas of cell subsets in BHA and pDev-dECM-BHA groups. (B) Expression profile of *Itgav* and *Itgb5* across macrophage subsets. (C) Network diagram of enriched biological events related to multi organ re-development of mesenchymal cell subsets in BHA and pDev-dECM-BHA groups. (D) KEGG enrichment analysis of differential genes in mesenchymal cell subsets in BHA and pDev-dECM-BHA groups. (E) Quantitative analysis of CD68 and ITGAV&ITGB5 co-localization in rat periodontal defect at 7 days post-implantation (n=3 per group). (F) Multiplex immunofluorescence analysis of macrophage polarization in rat periodontal defects at day 7. Representative images show the co-localization of CD68, CD206, and CD86. Nuclei were stained with DAPI. (G) Quantitative analysis of the number of CD68^+^CD206^+^ and CD68^+^CD86^+^ cells (n=5 per group). (H) IHC staining images of FN1 and CD10 of rat periodontal defect after 7 days of material implantation. (I) Micro-CT based measurement of new bone formation in rat periodontal defect at 4 weeks post-implantation (n=4 per group). (J) Quantitative evaluation of collagen regeneration in rat periodontal defect at 4 weeks using polarized Sirius Red staining (n=5 per group). Data were presented as mean ± SD by one-way ANOVA with Tukey’s post hoc test. **P* < 0.05, ***P* < 0.01, ****P* < 0.001, *****P* < 0.0001.

Supplementary Table 1. RT-qPCR primers applied in this study

| Genes | Primer sequences (5’-3’) | |
| --- | --- | --- |
| *g-Gapdh* | Forward:  Reverse: | TCAGCAATGCCTCCTGCAC  TCTGGGTGGCAGTGATGGC |
| *m-Mfge8* | Forward: | AGATGCGGGTATCAGGTGTGA |
|  | Reverse: | GGGGCTCAGAACATCCGTG |
| *m-Itgb5* | Forward: | CTTACCCTGGTCAGAGGAAGTG |
|  | Reverse: | CCTCAAGGTGAAAGACTGTGCTG |
| *m-Itgav* | Forward: | TTGATTCAACAGGCAATCGAGA |
|  | Reverse: | AGCATACTCAACGGTCTTTGTG |
| *m-Cd36* | Forward: | ATGGGCTGTGATCGGAACT |
|  | Reverse: | GTCTTCCCAATAAGCATGTCTCC |
| *m-Stab1* | Forward: | GGACTGCCGCTATGAGTTG |
|  | Reverse: | GGACTGCCGCTATGAGTTG |
| *m-Axl* | Forward: | GGAACCCAGGGAATATCACAGG |
|  | Reverse: | AGTTCTAGGATCTGTCCATCTCG |
| *m-Il1b* | Forward: | TCTGTGACTCGTGGGATGAT |
|  | Reverse: | CTTCTTTGGGTATTGTTTGG |
| *m-Tgfb1* | Forward: | GTGGAAATCAACGGGATCAGC |
|  | Reverse: | CAGCAGTTCTTCTCTGTGGAGC |
| *m-Arg1* | Forward: | GGAATCTGCATGGGCAACCTGTGT |
|  | Reverse: | AGGGTCTACGTCTCGCAAGCCA |
| *m-Il10* | Forward: | TCCGGGGTGACAATAACTGC |
|  | Reverse: | TGGCCTTGTAGACACCTTTGT |

Supplementary Table 2. The top 20 differentially expressed genes and selected functional markers for macrophage subpopulations during periodontal development, maturation, and repair

| Cluster | Gene  Symbol | Avg_  log2FC | P_value | Annotation Note |
| --- | --- | --- | --- | --- |
| Macro_Dev | *Egr1* | 2.65 | 1.04E-49 | Top 20 |
| Macro_Dev | *Ptprm* | 2.64 | 6.93E-57 | Top 20 |
| Macro_Dev | *Mertk* | 2.46 | 7.73E-78 | Top 20/Selected Functional Marker |
| Macro_Dev | *Slc9a9* | 2.38 | 7.66E-55 | Top 20 |
| Macro_Dev | *Gdf15* | 2.37 | 1.57E-43 | Top 20 |
| Macro_Dev | *Jun* | 2.32 | 5.68E-48 | Top 20 |
| Macro_Dev | *Chka* | 2.27 | 2.76E-46 | Top 20 |
| Macro_Dev | *Tmcc3* | 2.18 | 2.67E-63 | Top 20 |
| Macro_Dev | *Atxn1* | 2.17 | 7.56E-65 | Top 20 |
| Macro_Dev | *Frmd4b* | 2.11 | 2.06E-66 | Top 20 |
| Macro_Dev | *Tanc2* | 2.07 | 1.32E-75 | Top 20 |
| Macro_Dev | *Zdhhc14* | 2.04 | 1.37E-70 | Top 20 |
| Macro_Dev | *Tcf7l2* | 2.04 | 3.30E-53 | Top 20 |
| Macro_Dev | *Pitpnc1* | 2.00 | 8.25E-50 | Top 20 |
| Macro_Dev | *Dip2c* | 1.97 | 8.47E-74 | Top 20 |
| Macro_Dev | *Wdfy3* | 1.94 | 2.31E-53 | Top 20 |
| Macro_Dev | *Dst* | 1.91 | 2.15E-57 | Top 20 |
| Macro_Dev | *Id2* | 1.86 | 5.88E-32 | Top 20 |
| Macro_Dev | *Cxcl2* | 1.84 | 2.06E-20 | Top 20 |
| Macro_Dev | *Snx24* | 1.83 | 7.65E-67 | Top 20 |
| Macro_Dev | *Abca1* | 1.46 | 6.82E-39 | Selected Functional Marker |
| Macro_Dev | *Mrc1* | 1.05 | 7.48E-35 | Selected Functional Marker |
| Macro_Dev | *Il10* | 0.80 | 5.92E-05 | Selected Functional Marker |
| Macro_Pre_Dev | *Hbb-bs* | 2.40 | 1.58E-66 | Top 20 |
| Macro_Pre_Dev | *Hbb-bt* | 2.33 | 7.63E-39 | Top 20 |
| Macro_Pre_Dev | *Prxl2b* | 2.31 | 3.08E-18 | Top 20 |
| Macro_Pre_Dev | *Hba-a1* | 2.31 | 4.26E-45 | Top 20 |
| Macro_Pre_Dev | *Blvrb* | 2.16 | 2.90E-22 | Top 20 |
| Macro_Pre_Dev | *Serpinb6a* | 2.05 | 2.52E-20 | Top 20 |
| Macro_Pre_Dev | *Hba-a2* | 1.98 | 5.75E-33 | Top 20 |
| Macro_Pre_Dev | *Fcna* | 1.97 | 1.91E-13 | Top 20 |
| Macro_Pre_Dev | *Ftl1* | 1.84 | 6.09E-59 | Top 20 |
| Macro_Pre_Dev | *Hebp1* | 1.83 | 1.78E-09 | Top 20 |
| Macro_Pre_Dev | *Il18* | 1.82 | 1.84E-07 | Top 20 |
| Macro_Pre_Dev | *Txn1* | 1.82 | 5.87E-14 | Top 20 |
| Macro_Pre_Dev | *C1qb* | 1.77 | 1.49E-41 | Top 20 |
| Macro_Pre_Dev | *C1qc* | 1.75 | 3.79E-40 | Top 20 |
| Macro_Pre_Dev | *Aif1* | 1.75 | 4.74E-20 | Top 20 |
| Macro_Pre_Dev | *Cox5a* | 1.65 | 7.19E-19 | Top 20 |
| Macro_Pre_Dev | *Fabp5* | 1.65 | 1.75E-07 | Top 20 |
| Macro_Pre_Dev | *C1qa* | 1.63 | 8.21E-38 | Top 20 |
| Macro_Pre_Dev | *Hmox1* | 1.63 | 9.18E-14 | Top 20 |
| Macro_Pre_Dev | *Pld3* | 1.61 | 2.25E-07 | Top 20 |
| Macro_Pre_Dev | *Cd163* | 1.24 | 2.56E-02 | Selected Functional Marker |
| Macro_Pre_Dev | *Cd68* | 1.22 | 1.94E-01 | Selected Functional Marker |
| Macro_Re | *S100a4* | 3.03 | 5.39E-94 | Top 20 |
| Macro_Re | *Cytip* | 2.84 | 7.50E-97 | Top 20 |
| Macro_Re | *H2-Ab1* | 2.81 | 6.21E-81 | Top 20 |
| Macro_Re | *Alcam* | 2.76 | 1.16E-85 | Top 20 |
| Macro_Re | *Plac8* | 2.70 | 2.47E-69 | Top 20 |
| Macro_Re | *Ifitm3* | 2.69 | 1.36E-76 | Top 20 |
| Macro_Re | *Il1b* | 2.68 | 2.32E-59 | Top 20/Selected Functional Marker |
| Macro_Re | *Cd209a* | 2.66 | 4.57E-67 | Top 20 |
| Macro_Re | *H2-Aa* | 2.65 | 1.46E-77 | Top 20 |
| Macro_Re | *Xylt1* | 2.64 | 4.32E-93 | Top 20 |
| Macro_Re | *Cd74* | 2.55 | 3.50E-76 | Top 20/Selected Functional Marker |
| Macro_Re | *H2-Eb1* | 2.50 | 9.02E-74 | Top 20 |
| Macro_Re | *S100a6* | 2.50 | 1.31E-84 | Top 20 |
| Macro_Re | *Napsa* | 2.45 | 3.51E-100 | Top 20 |
| Macro_Re | *Gpr132* | 2.38 | 2.09E-96 | Top 20 |
| Macro_Re | *Lsp1* | 2.36 | 4.60E-87 | Top 20 |
| Macro_Re | *Fxyd5* | 2.31 | 3.83E-84 | Top 20 |
| Macro_Re | *Ccr2* | 2.31 | 8.48E-92 | Top 20 |
| Macro_Re | *Etv6* | 2.20 | 1.08E-74 | Top 20 |
| Macro_Re | *Malt1* | 2.18 | 1.24E-55 | Top 20 |

Supplementary Table 3. The top 20 differentially expressed genes and selected functional markers for macrophage subpopulations in regenerative periodontal defects

| Cluster | Gene  Symbol | Avg_  log2FC | P_value | Annotation Note |
| --- | --- | --- | --- | --- |
| Macro_Dev | *Ctsd* | 2.42 | 1.85E-242 | Top 20 |
| Macro_Dev | *Rarres1* | 2.31 | 3.32E-199 | Top 20 |
| Macro_Dev | *Ctsl* | 2.22 | 2.28E-201 | Top 20 |
| Macro_Dev | *Gpnmb* | 2.17 | 7.00E-212 | Top 20 |
| Macro_Dev | *Ctsb* | 2.05 | 7.70E-220 | Top 20 |
| Macro_Dev | *Lgmn* | 1.91 | 1.68E-228 | Top 20 |
| Macro_Dev | *Apoe* | 1.87 | 3.62E-144 | Top 20 |
| Macro_Dev | *Cd8a* | 1.84 | 7.58E-150 | Top 20 |
| Macro_Dev | *Gsta1* | 1.77 | 4.73E-77 | Top 20 |
| Macro_Dev | *Stab1* | 1.76 | 3.68E-163 | Top 20 |
| Macro_Dev | *Pf4* | 1.76 | 7.33E-158 | Top 20 |
| Macro_Dev | *Slc9a9* | 1.73 | 8.98E-141 | Top 20 |
| Macro_Dev | *Trem2* | 1.73 | 2.77E-187 | Top 20 |
| Macro_Dev | *Hmox1* | 1.71 | 5.98E-101 | Top 20 |
| Macro_Dev | *Colec12* | 1.70 | 4.39E-173 | Top 20 |
| Macro_Dev | *Smpdl3a* | 1.66 | 3.17E-172 | Top 20 |
| Macro_Dev | *Cndp2* | 1.65 | 1.10E-183 | Top 20 |
| Macro_Dev | *Slc48a1* | 1.49 | 2.18E-132 | Top 20 |
| Macro_Dev | *Timp2* | 1.45 | 1.70E-196 | Top 20 |
| Macro_Dev | *Maf* | 1.41 | 8.88E-141 | Top 20 |
| Macro_Dev | *Mrc1* | 1.41 | 3.00E-147 | Selected Functional Marker |
| Macro_Dev | *Abca1* | 1.17 | 1.95E-91 | Selected Functional Marker |
| Macro_Dev | *Cd68* | 0.81 | 1.64E-96 | Selected Functional Marker |
| Macro_Dev | *Cd163* | 0.73 | 4.66E-24 | Selected Functional Marker |
| Macro_Dev | *Mertk* | 0.65 | 1.33E-64 | Selected Functional Marker |
| Macro_Pre_Dev | *Ccl4* | 3.02 | 3.64E-36 | Top 20 |
| Macro_Pre_Dev | *Cxcl10* | 2.81 | 2.14E-30 | Top 20 |
| Macro_Pre_Dev | *Oasl* | 2.61 | 2.65E-47 | Top 20 |
| Macro_Pre_Dev | *Hspa1b* | 2.61 | 2.56E-18 | Top 20 |
| Macro_Pre_Dev | *Cxcl13* | 2.29 | 3.84E-66 | Top 20 |
| Macro_Pre_Dev | *Selenop* | 2.01 | 2.06E-44 | Top 20 |
| Macro_Pre_Dev | *Cxcl2* | 1.96 | 3.73E-45 | Top 20 |
| Macro_Pre_Dev | *Cst3* | 1.90 | 9.79E-66 | Top 20 |
| Macro_Pre_Dev | *Phlda1* | 1.88 | 1.92E-43 | Top 20 |
| Macro_Pre_Dev | *Cfd* | 1.83 | 9.25E-28 | Top 20 |
| Macro_Pre_Dev | *Ccl3* | 1.83 | 2.49E-20 | Top 20 |
| Macro_Pre_Dev | *C1qc* | 1.80 | 2.94E-62 | Top 20 |
| Macro_Pre_Dev | *Jund* | 1.73 | 6.01E-68 | Top 20 |
| Macro_Pre_Dev | *C1qb* | 1.55 | 2.22E-51 | Top 20 |
| Macro_Pre_Dev | *Ccl24* | 1.54 | 1.98E-35 | Top 20 |
| Macro_Pre_Dev | *LOC102546903* | 1.53 | 1.92E-59 | Top 20 |
| Macro_Pre_Dev | *Ubc* | 1.53 | 1.30E-73 | Top 20 |
| Macro_Pre_Dev | *Fcmr* | 1.49 | 4.98E-52 | Top 20 |
| Macro_Pre_Dev | *Ifit3* | 1.48 | 2.43E-20 | Top 20 |
| Macro_Pre_Dev | *Map6* | 1.43 | 1.80E-36 | Top 20 |
| Macro_Pre_Dev | *Il1b* | 1.08 | 1.05E-12 | Selected Functional Marker |
| Macro_Pre_Dev | *Il10* | 2.20 | 2.63E-03 | Selected Functional Marker |
| Macro_Pre_Dev | *Cd74* | 0.55 | 2.33E-06 | Selected Functional Marker |
| Macro_Pre_Dev | *Cd68* | 0.25 | 7.77E-07 | Selected Functional Marker |
| Macro_Re | *Plac8* | 2.95 | 1.01E-110 | Top 20 |
| Macro_Re | *Malt1* | 2.95 | 1.26E-184 | Top 20 |
| Macro_Re | *RT1-Ba* | 2.94 | 2.25E-259 | Top 20 |
| Macro_Re | *Napsa* | 2.86 | 4.70E-220 | Top 20 |
| Macro_Re | *RT1-Da* | 2.83 | 2.19E-247 | Top 20 |
| Macro_Re | *RT1-Db1* | 2.79 | 4.55E-244 | Top 20 |
| Macro_Re | *RT1-Bb* | 2.73 | 2.40E-227 | Top 20 |
| Macro_Re | *Cdc14a* | 2.67 | 3.21E-141 | Top 20 |
| Macro_Re | *Ccl17* | 2.57 | 1.28E-84 | Top 20 |
| Macro_Re | *Cd74* | 2.42 | 9.48E-233 | Top 20/Selected Functional Marker |
| Macro_Re | *Errfi1* | 2.37 | 5.62E-96 | Top 20 |
| Macro_Re | *Lsp1* | 2.36 | 4.37E-224 | Top 20 |
| Macro_Re | *Nr4a3* | 2.30 | 2.98E-108 | Top 20 |
| Macro_Re | *Slco3a1* | 2.30 | 9.80E-139 | Top 20 |
| Macro_Re | *Tbc1d4* | 2.27 | 1.11E-27 | Top 20 |
| Macro_Re | *Cytip* | 2.00 | 3.15E-140 | Top 20 |
| Macro_Re | *Ccr2* | 1.96 | 4.91E-152 | Top 20 |
| Macro_Re | *Arhgap6* | 1.96 | 4.10E-82 | Top 20 |
| Macro_Re | *Cpne8* | 1.88 | 1.23E-59 | Top 20 |
| Macro_Re | *Bhlhe40* | 1.88 | 1.21E-166 | Top 20 |
| Macro_Re | *Il1b* | 0.97 | 1.15E-37 | Selected Functional Marker |
