# Supplementary figures and images for: Postnatal Maxillofacial “Developing” Decellularized Extracellular Matrix Orchestrates Hierarchical Cross-Organ Regeneration via Macrophage Integrin αvβ5-Mediated Efferocytosis-Driven Developmental Recapitulation
Source: Research (Wash D C). 2026 Apr 15;9:1234. doi: 10.34133/research.1234 (PMC13080099; doi:10.34133/research.1234)

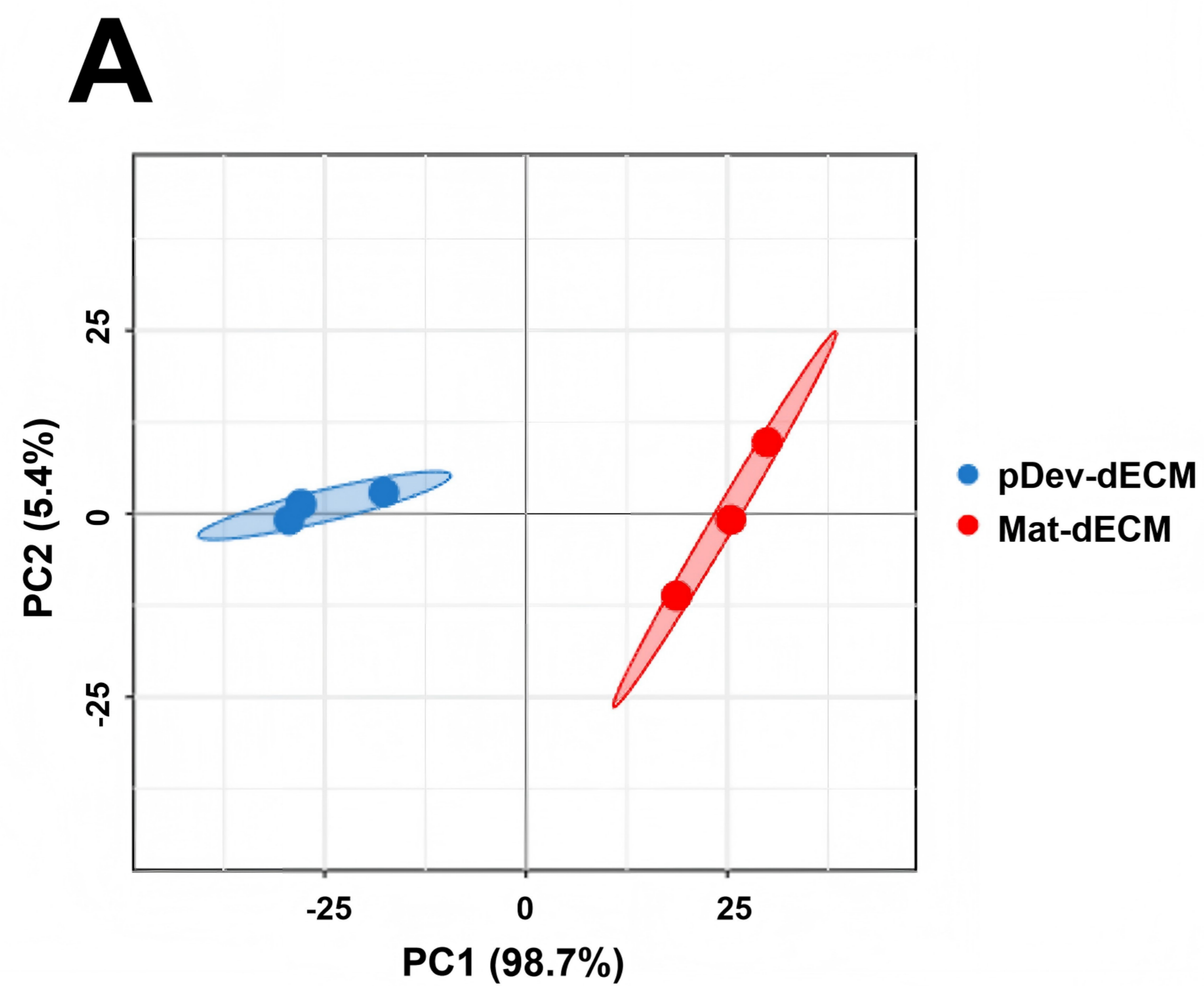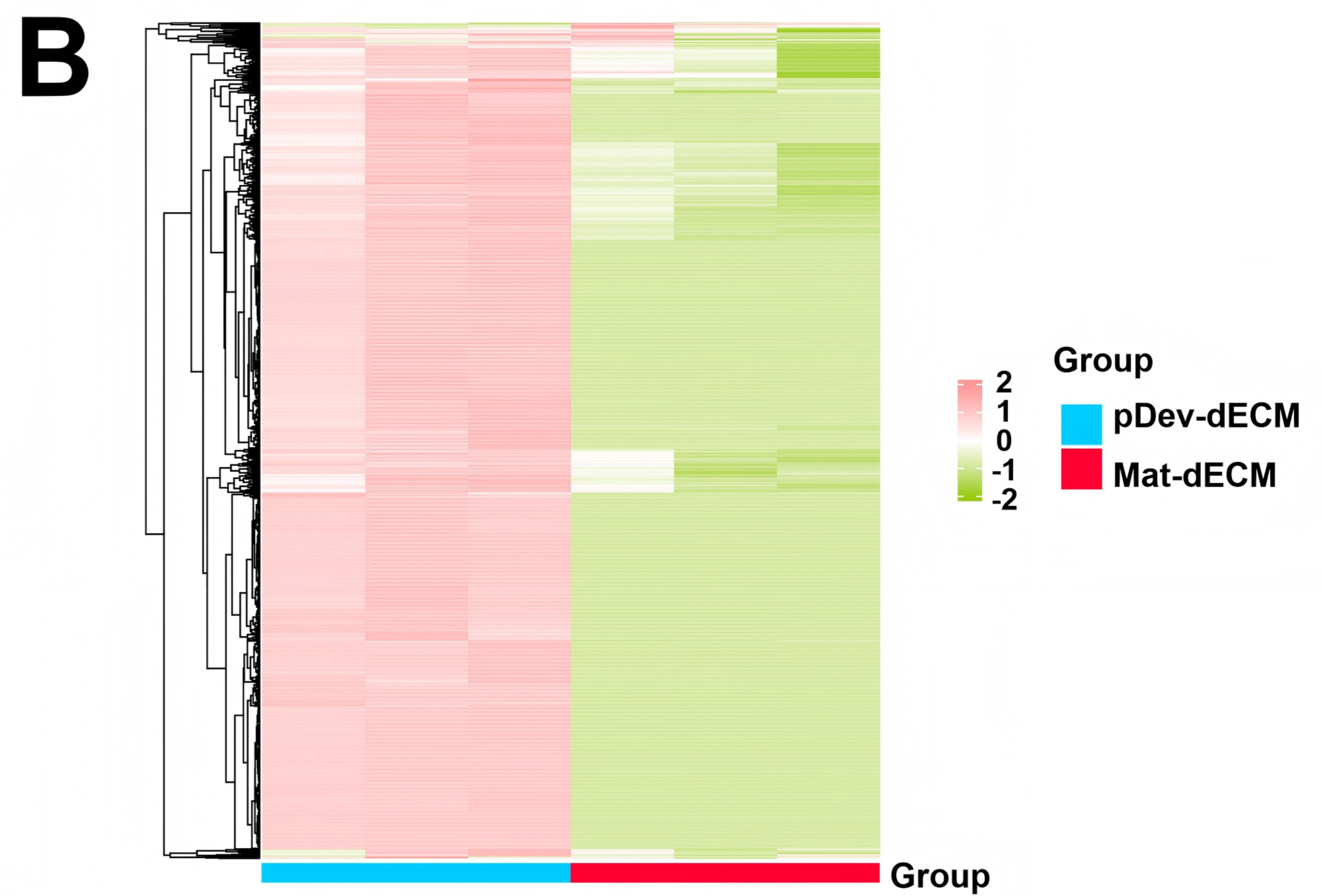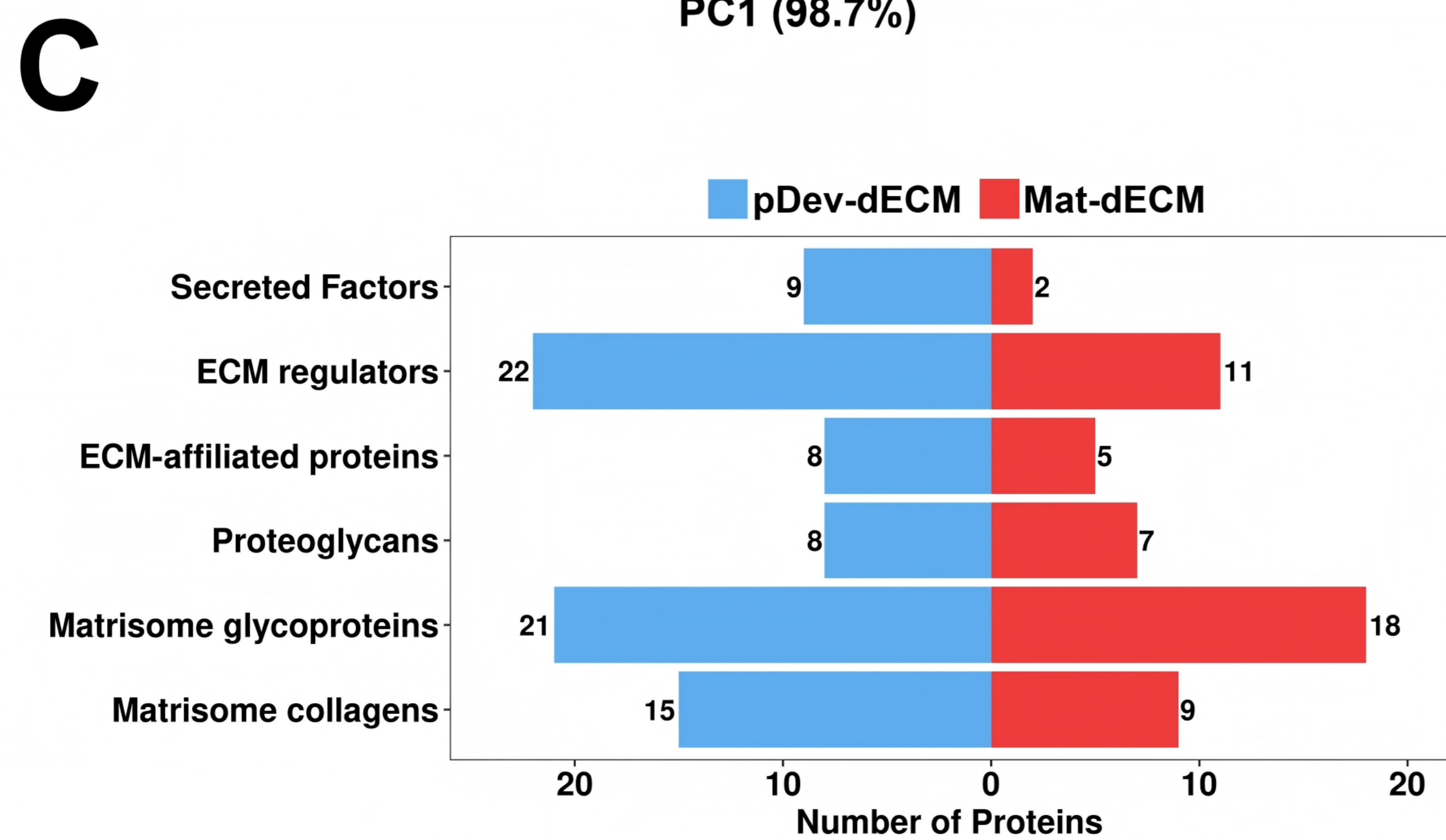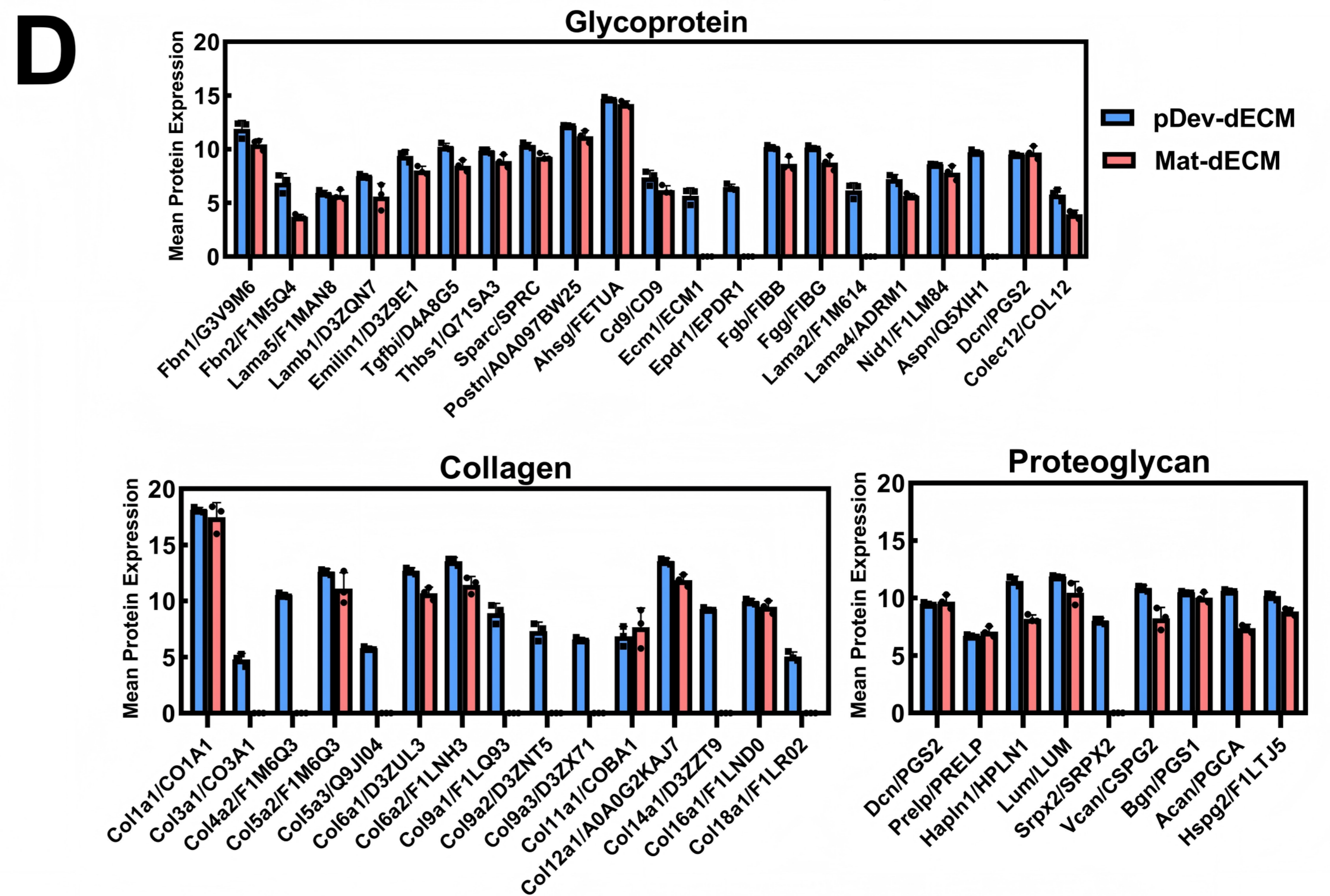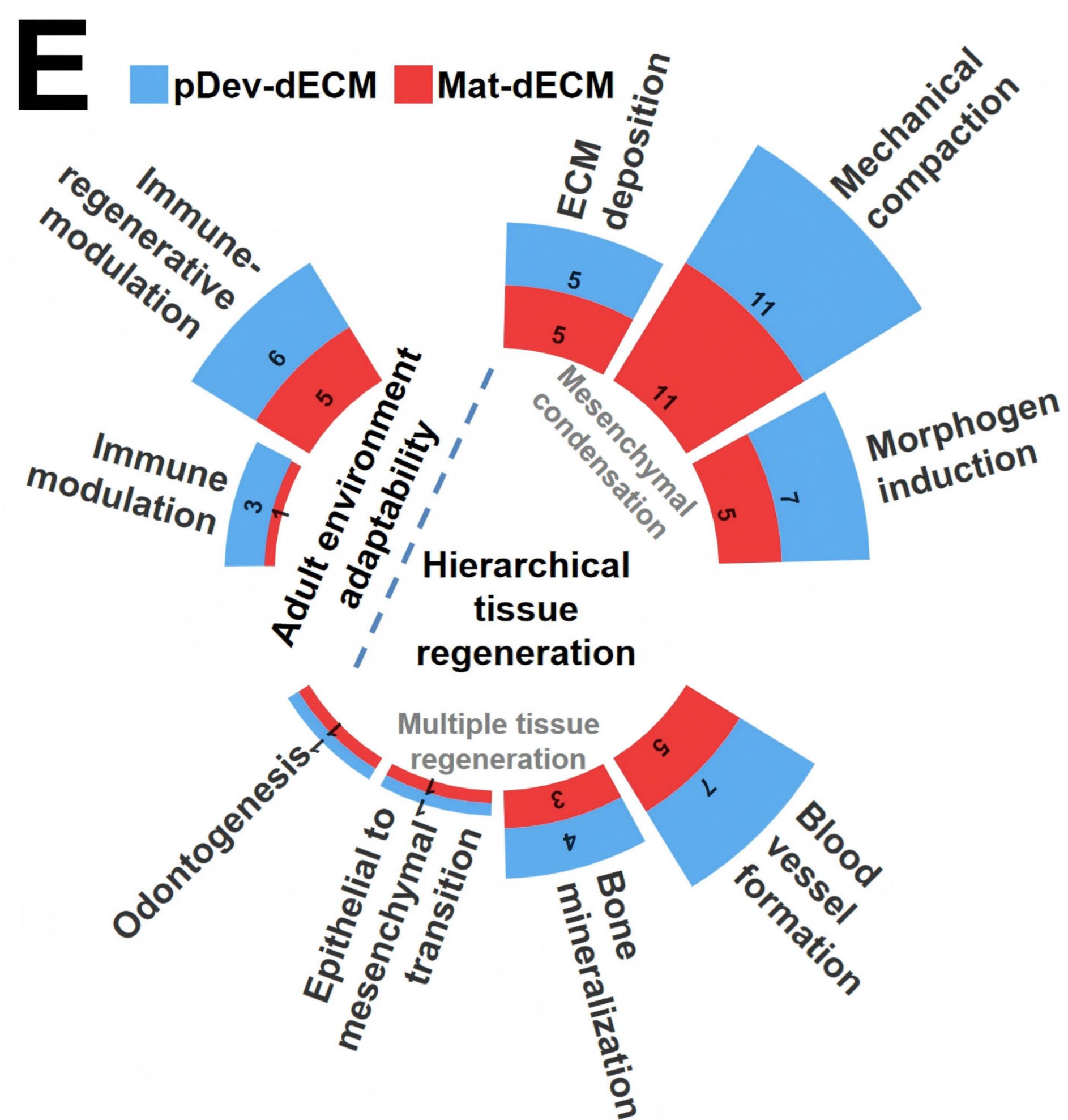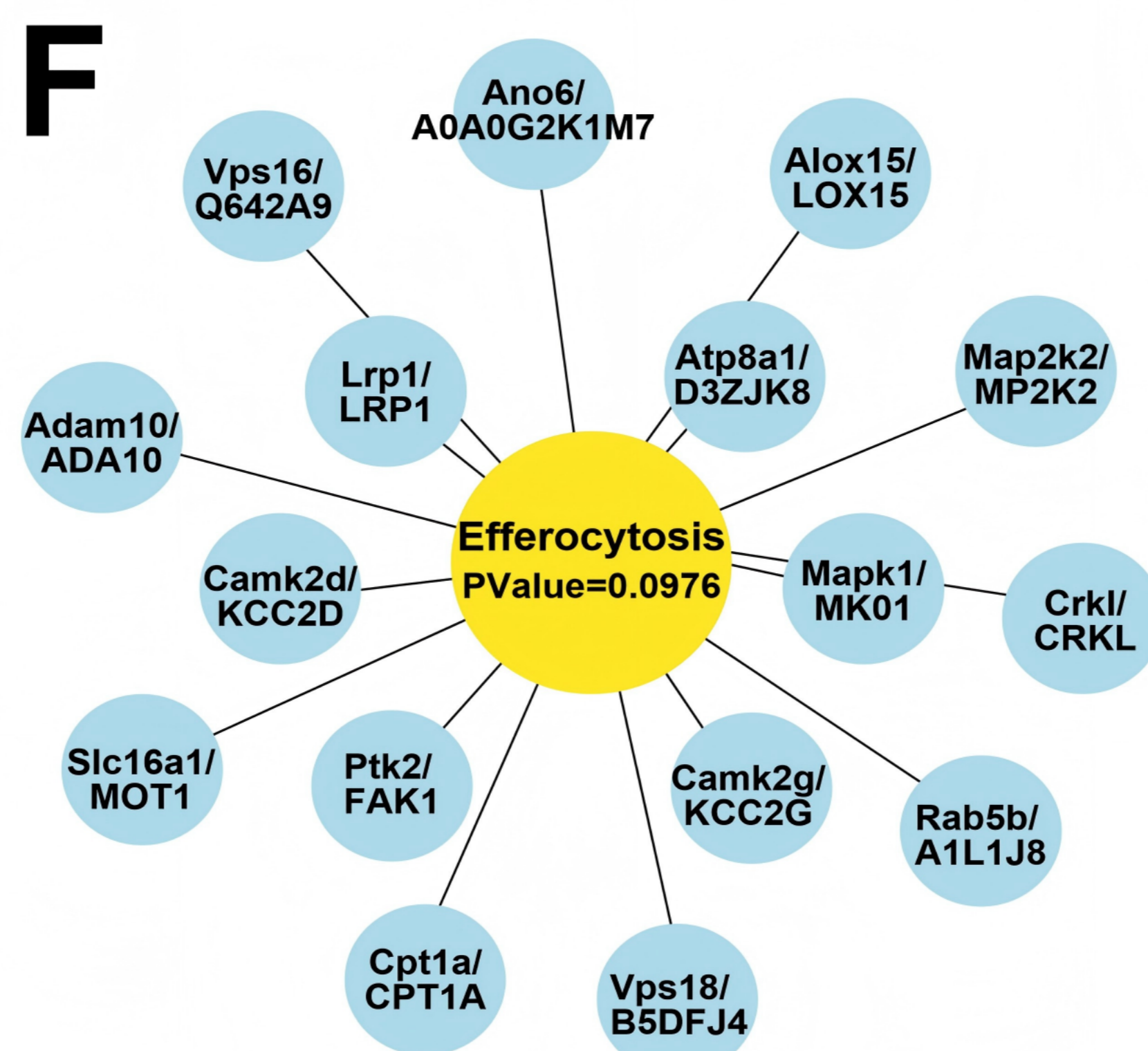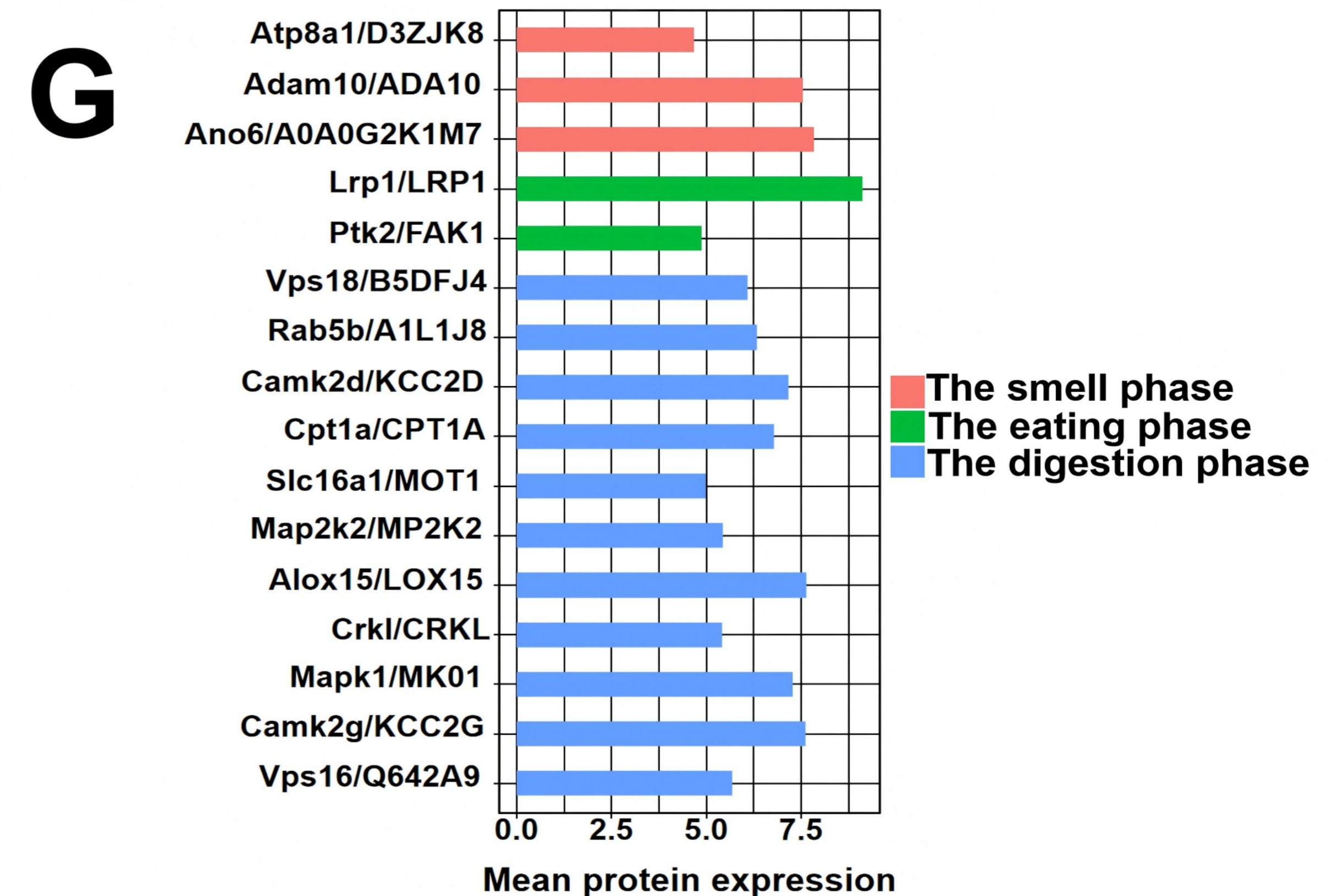

Supplement: Supplementary 1 — Figs. S1 to S5 Tables S1 to S3 [file research.1234.f1.zip › Supplementary Figure 1.pdf]

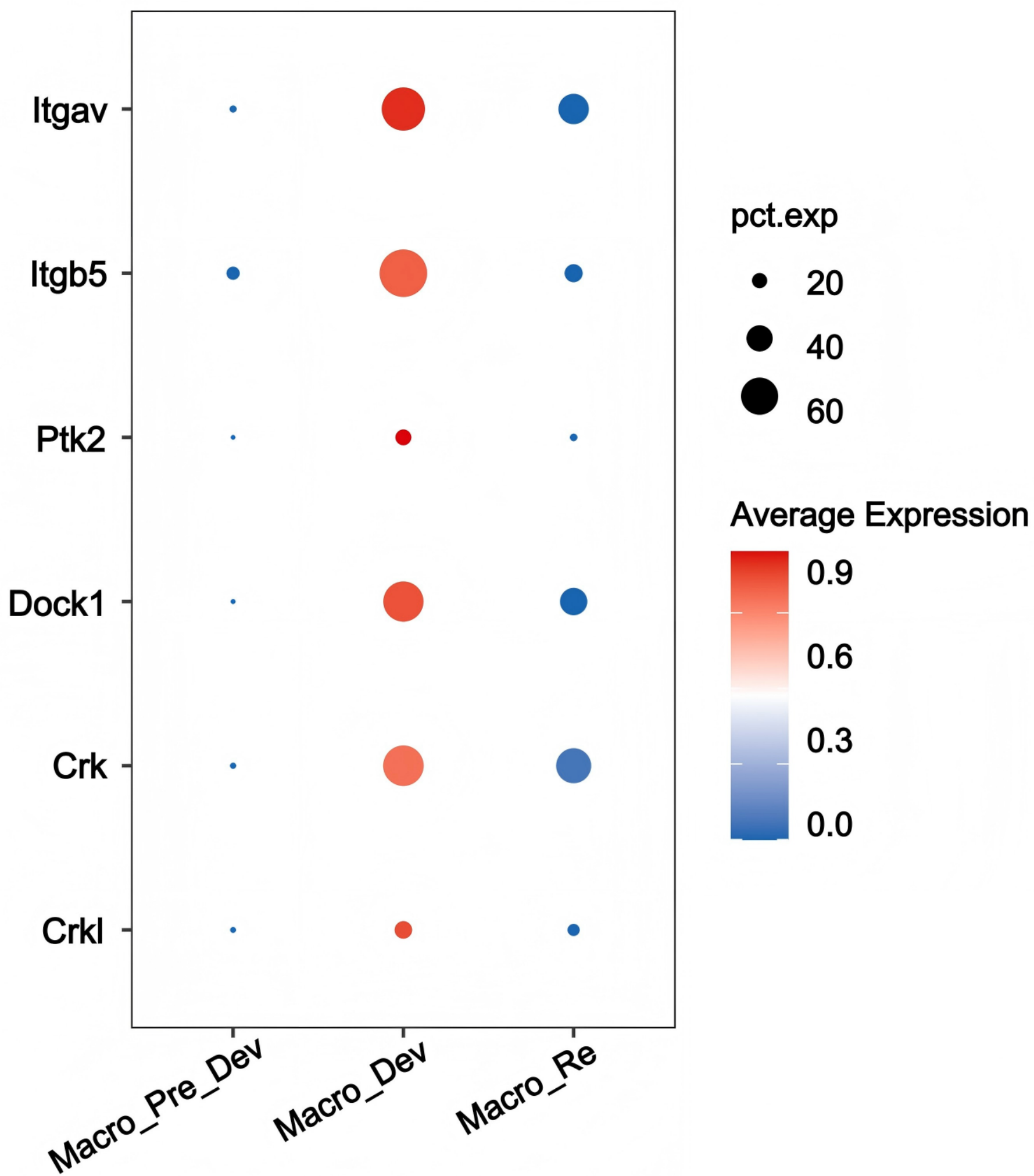

Supplement: Supplementary 1 — Figs. S1 to S5 Tables S1 to S3 [file research.1234.f1.zip › Supplementary Figure 2.pdf]

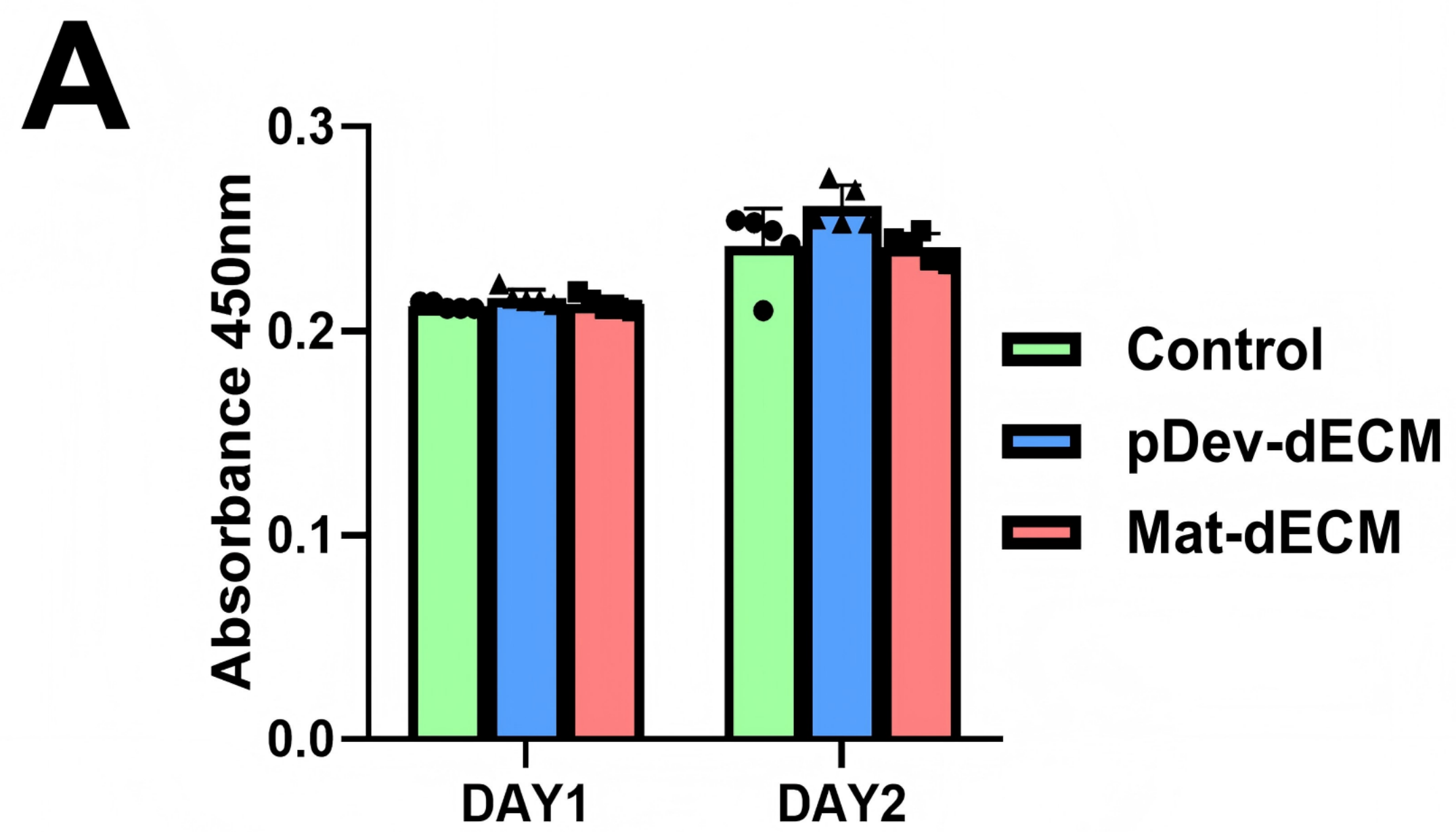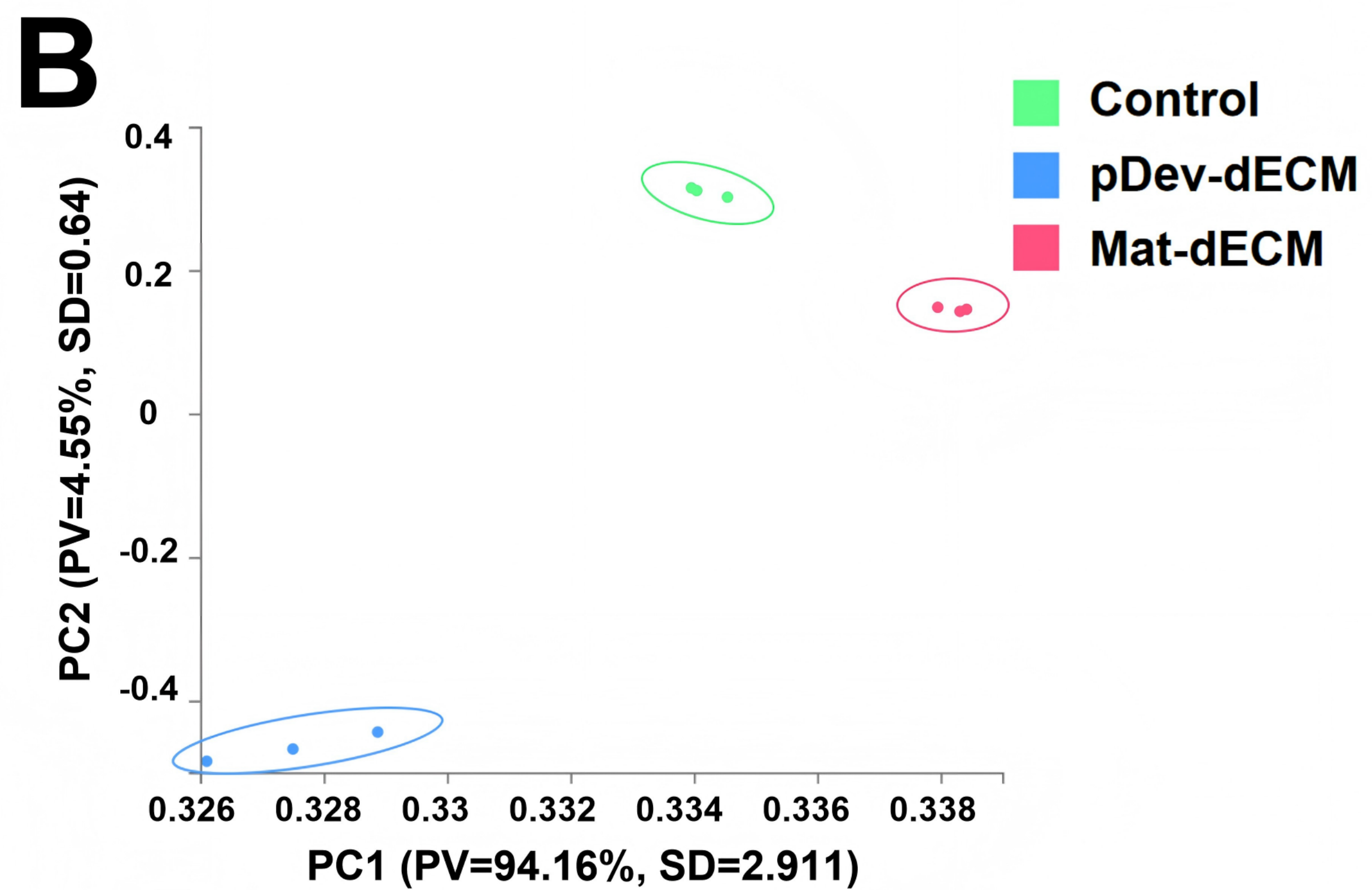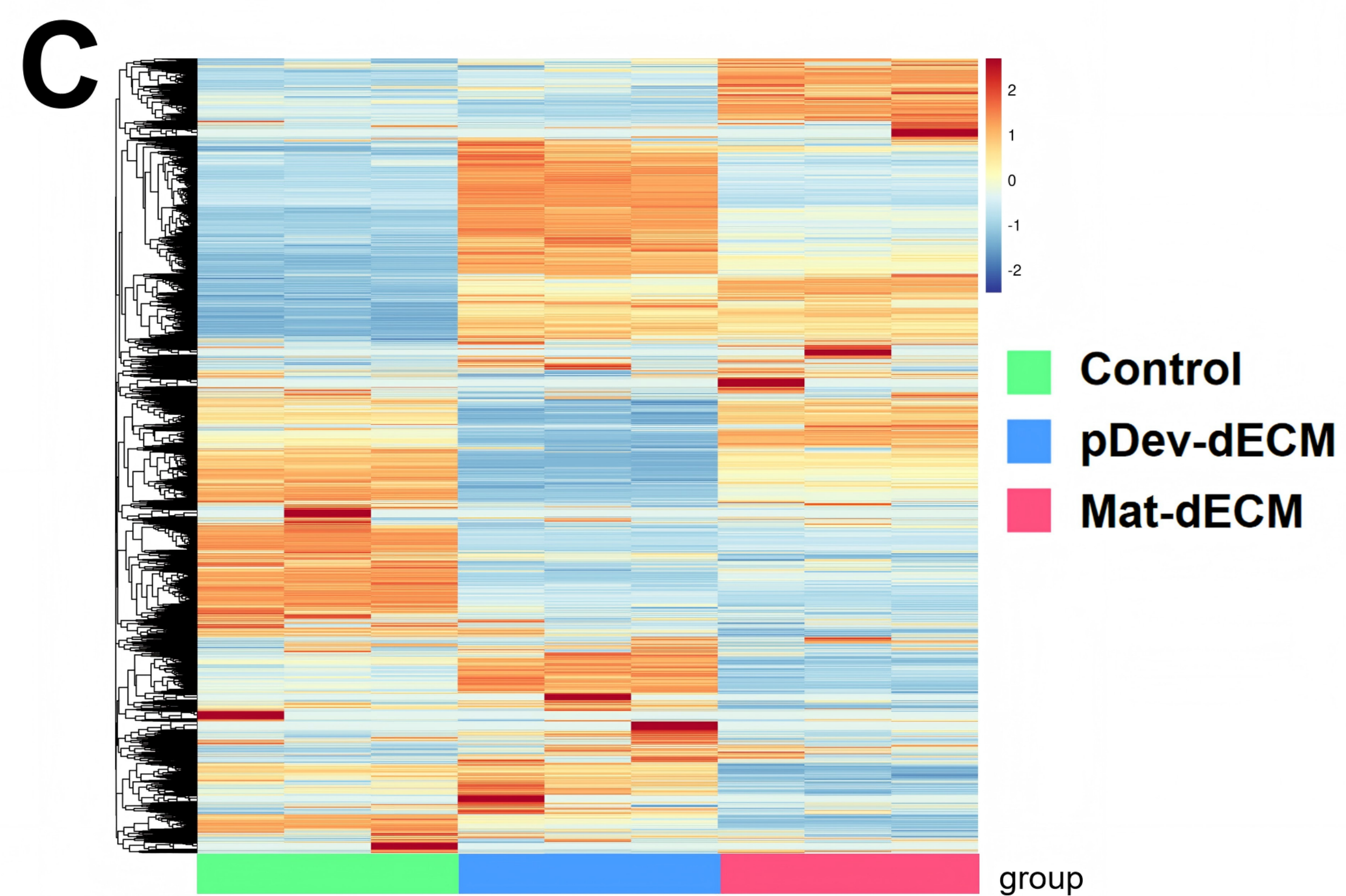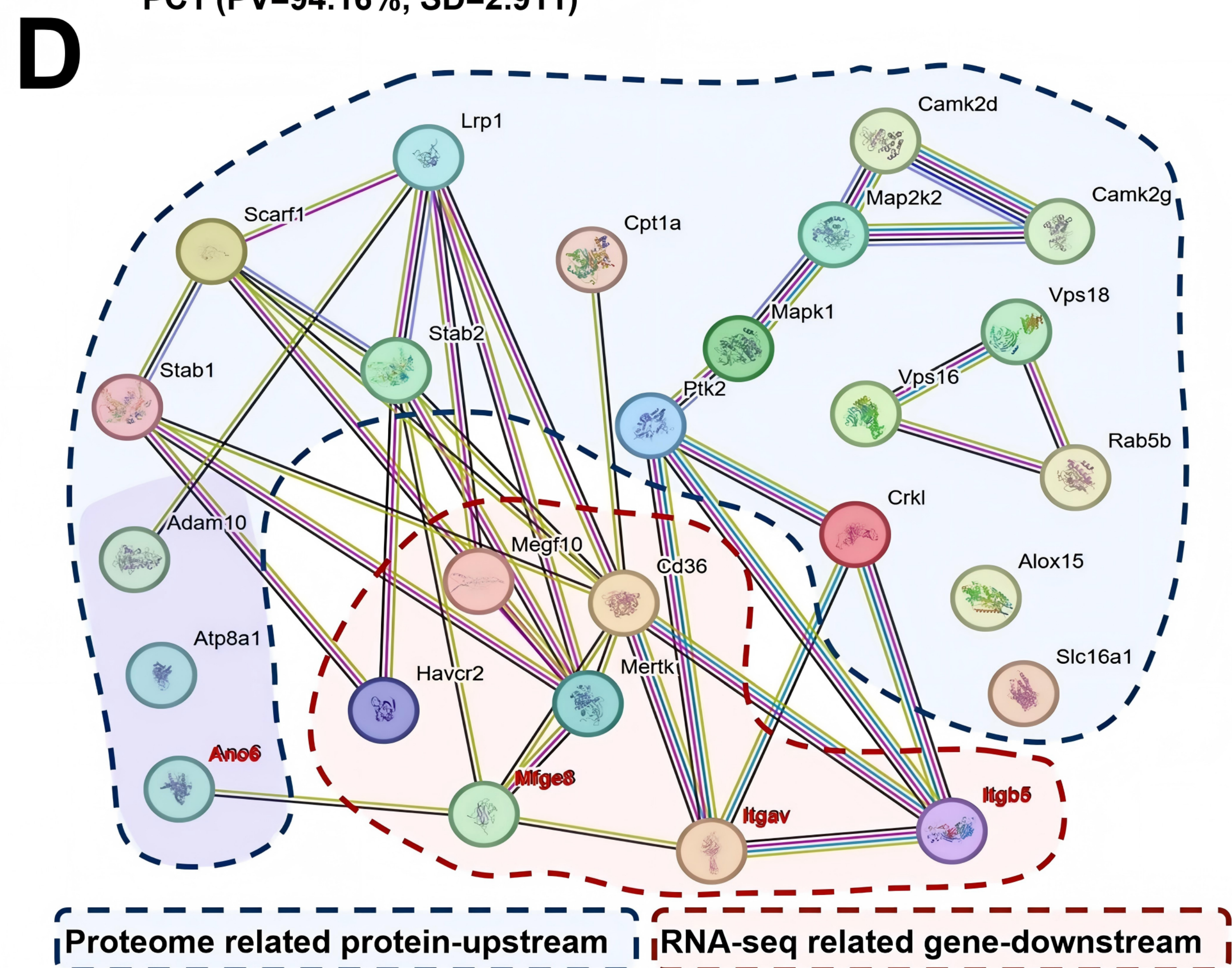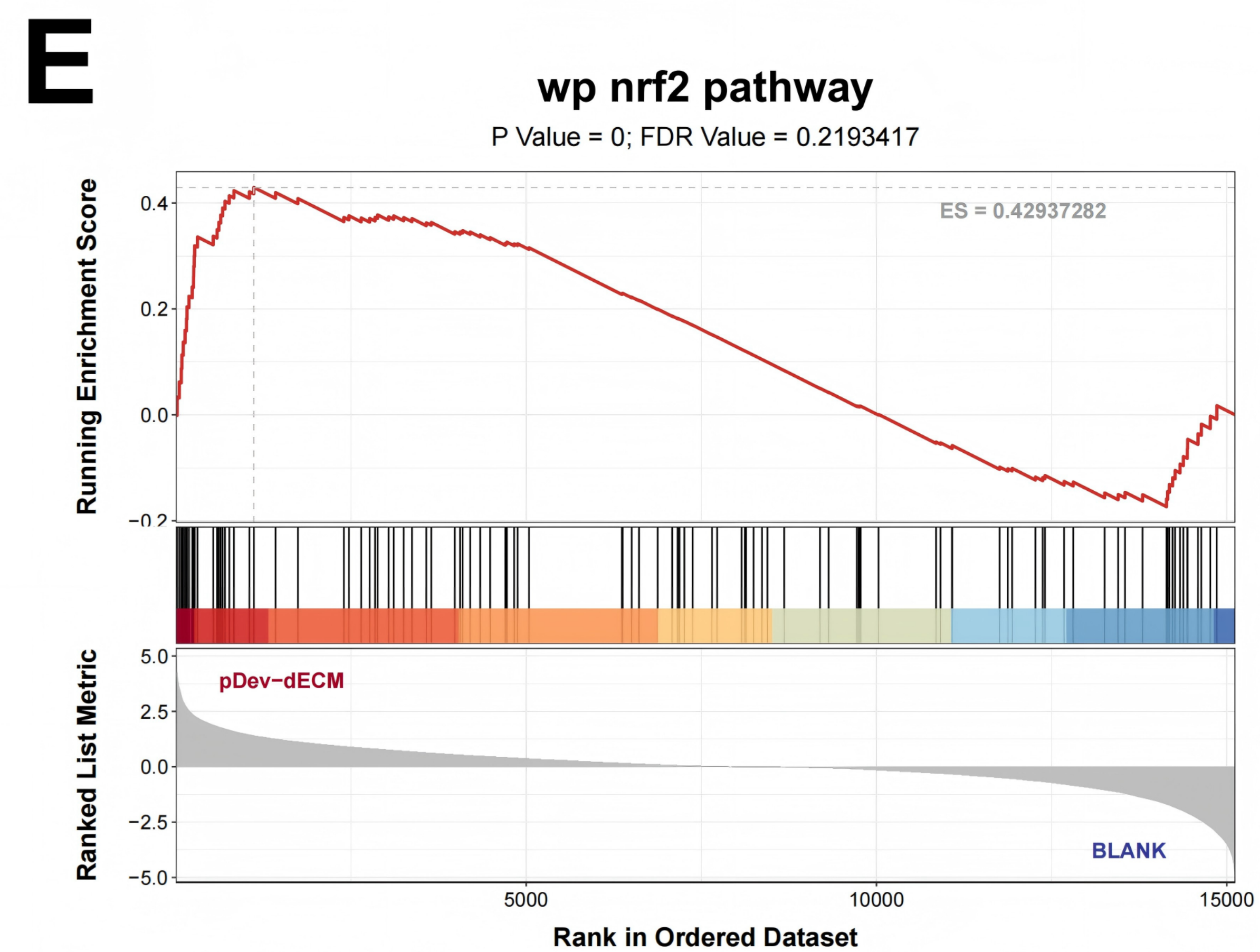

Supplement: Supplementary 1 — Figs. S1 to S5 Tables S1 to S3 [file research.1234.f1.zip › Supplementary Figure 3.pdf]

**A****BHA****pDev-dECM-BHA****Mat-dECM-BHA**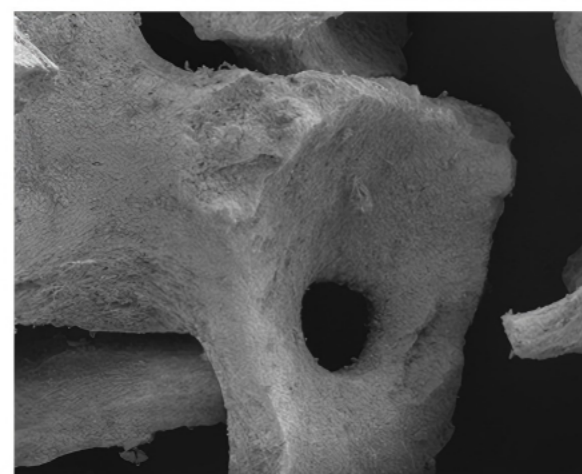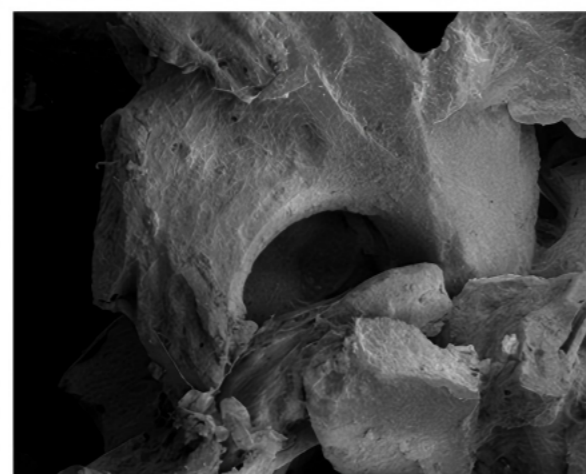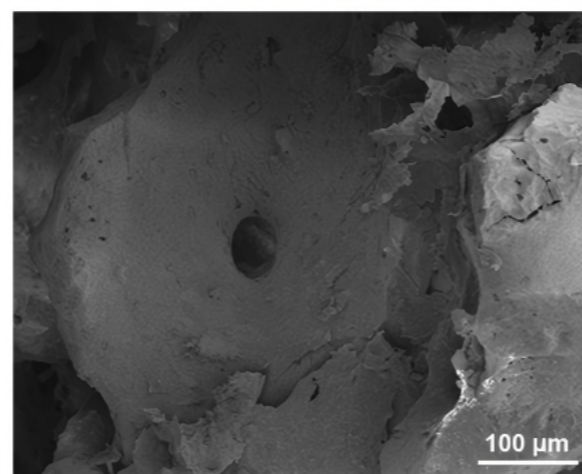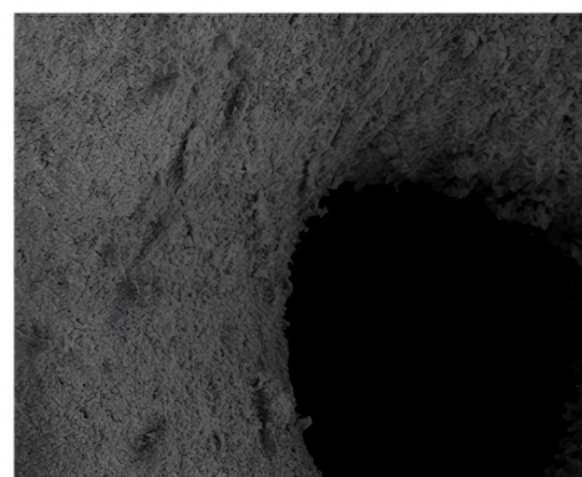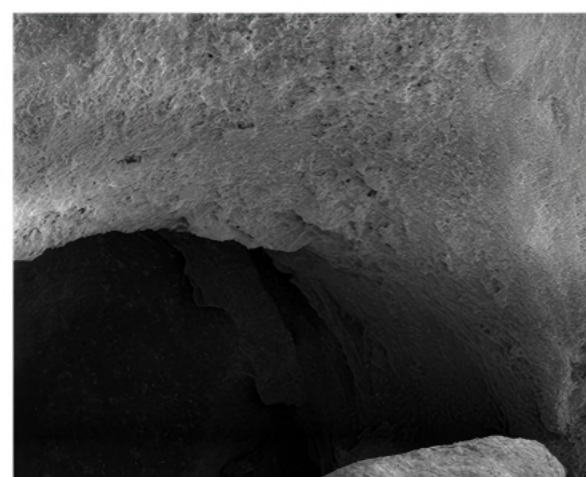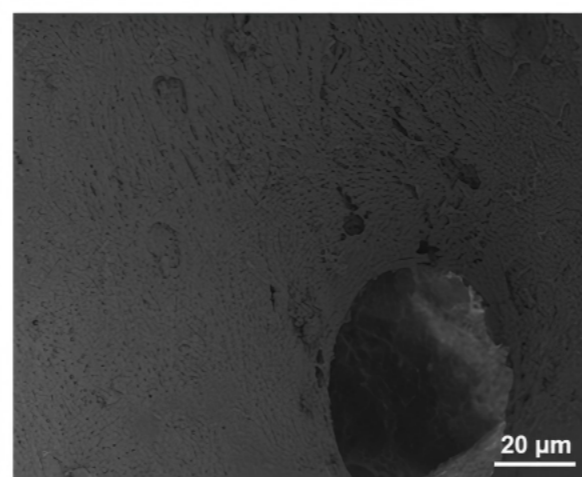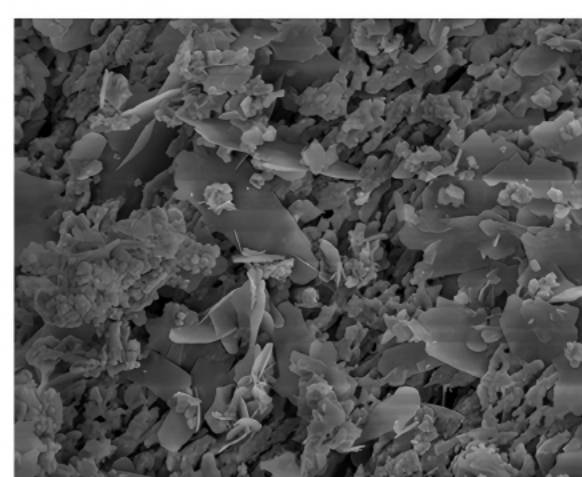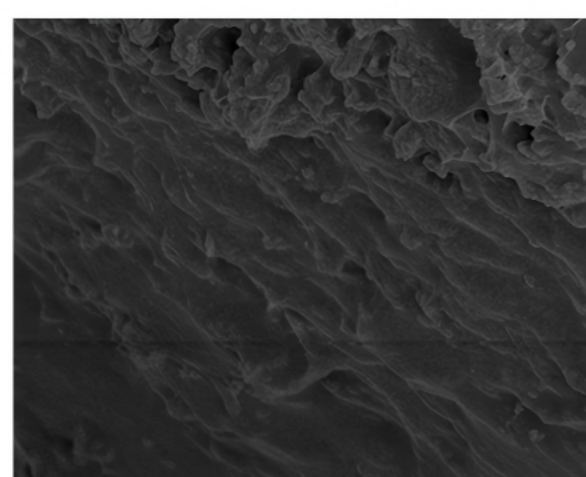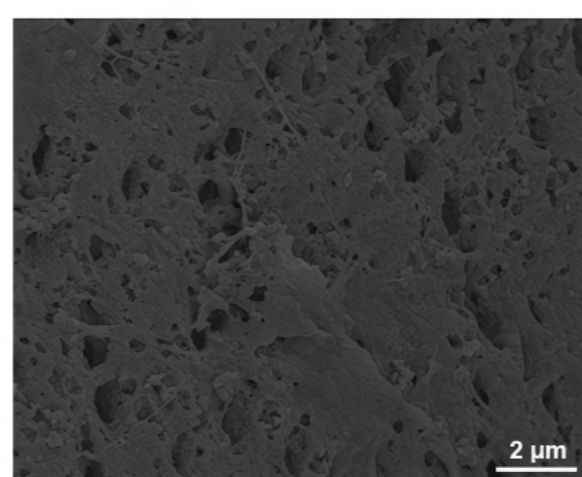**B**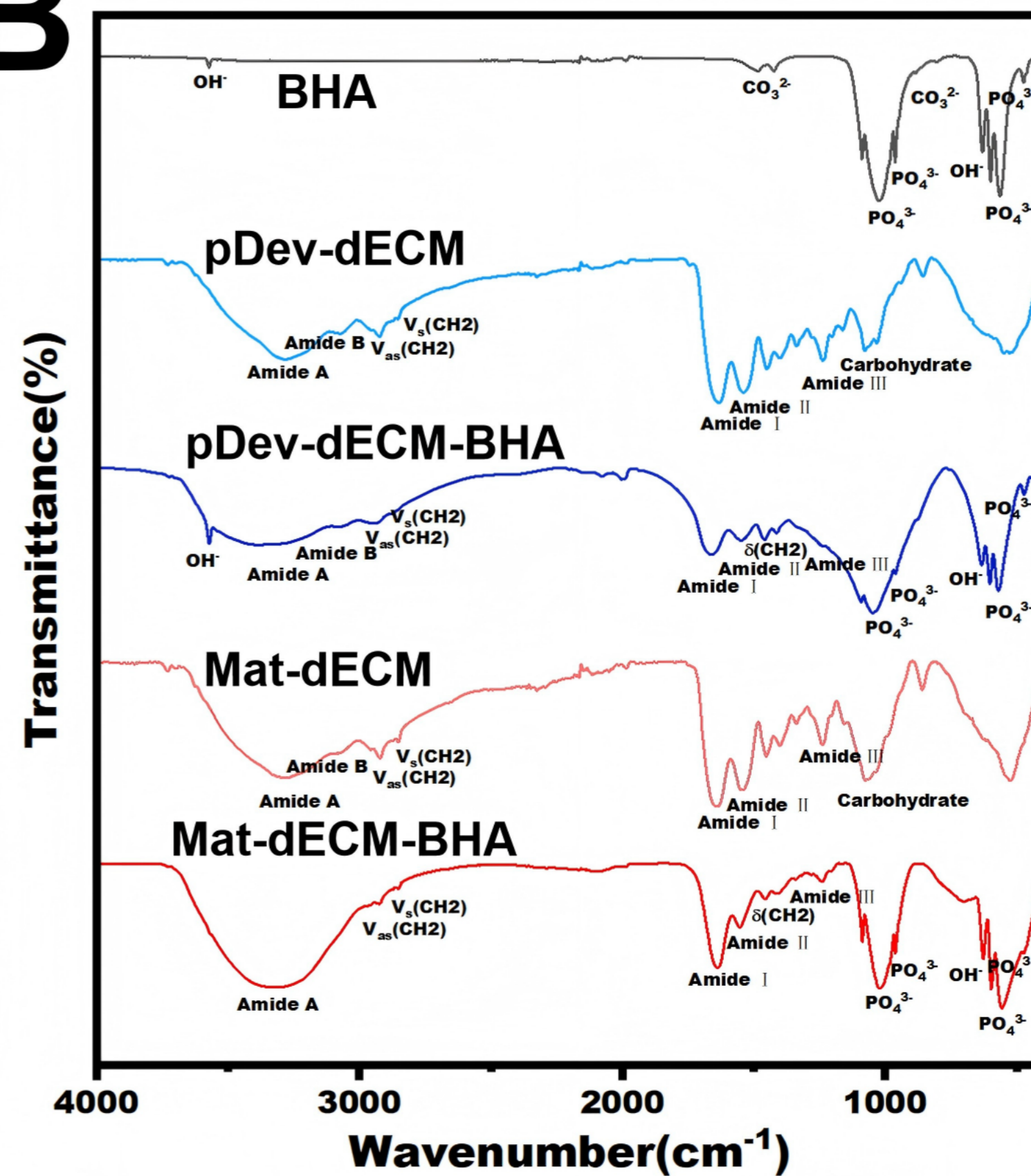

Supplement: Supplementary 1 — Figs. S1 to S5 Tables S1 to S3 [file research.1234.f1.zip › Supplementary Figure 4.pdf]
